# Maternal *Smc3* protects the integrity of the zygotic genome through DNA replication and mitosis

**DOI:** 10.1242/dev.199800

**Published:** 2021-12-22

**Authors:** Wei-Ting Yueh, Vijay Pratap Singh, Jennifer L. Gerton

**Affiliations:** 1 Stowers Institute for Medical Research, Kansas City, MO 64110, USA; 2 Department of Biochemistry and Molecular Biology, University of Kansas Medical Center, Kansas City, KS 66160, USA

**Keywords:** Cohesin, SMC3, Spontaneous DNA damage, Chromosome segregation, Micronuclei, Mouse, Zygote, Juvenile, Maternal-effect gene, Developmental competence

## Abstract

Aneuploidy is frequently observed in oocytes and early embryos, begging the question of how genome integrity is monitored and preserved during this crucial period. SMC3 is a subunit of the cohesin complex that supports genome integrity, but its role in maintaining the genome during this window of mammalian development is unknown. We discovered that, although depletion of *Smc3* following meiotic S phase in mouse oocytes allowed accurate meiotic chromosome segregation, adult females were infertile. We provide evidence that DNA lesions accumulated following S phase in SMC3-deficient zygotes, followed by mitosis with lagging chromosomes, elongated spindles, micronuclei, and arrest at the two-cell stage. Remarkably, although centromeric cohesion was defective, the dosage of SMC3 was sufficient to enable embryogenesis in juvenile mutant females. Our findings suggest that, despite previous reports of aneuploidy in early embryos, chromosome missegregation in zygotes halts embryogenesis at the two-cell stage. *Smc3* is a maternal gene with essential functions in the repair of spontaneous damage associated with DNA replication and subsequent chromosome segregation in zygotes, making cohesin a key protector of the zygotic genome.

## Abstract (precis)

**Summary:** This study demonstrates at the molecular level how maternally provided SMC3 supports the first round of DNA replication and chromosome segregation in the zygote to enable successful embryogenesis.

## INTRODUCTION

Infertility impacts the psychological well-being, economic status, mental health, sexual and marital relationships, and quality of life of women and couples (Luk and Loke, 2015; Namdar et al., 2017), with 4.65 million couples having experienced infertility in the USA in 2013 alone (Thoma et al., 2013). Among numerous factors, oocyte quality contributes significantly to infertility. Female reproduction is profoundly affected by age (Hassold and Hunt, 2001; Hunt, 2017); fertility starts to decline during the mid-thirties and accelerates thereafter. Women are born with all the eggs for their lifetime and these decrease in quality and quantity with age. Although faulty meiosis is a major contributor to female infertility, early embryonic failure also contributes, making it desirable to have visual markers of *in vitro* fertilization that indicate successful outcomes. Many factors essential for developmental competence are loaded into oocytes, so-called ‘maternal-effect genes’.

Chromosome segregation during early development in mammals is highly error prone and aneuploidy abounds in oocytes and early embryos (Hassold and Hunt, 2001; Vázquez-Diez and FitzHarris, 2018; Radonova et al., 2019; Pauerova et al., 2020), underscoring the need to understand how the genome is transmitted from one generation to the next. Cohesin is a genome maintenance protein complex required for successful meiosis and mitosis. It is a ring-shaped multiprotein complex that has multiple crucial functions in sister chromatid cohesion, DNA damage repair, higher-order chromosome structure and gene expression, including epigenetic reprogramming. The cohesin ring consists of four modular subunits: SMC1α or β, SMC3, RAD21 or REC8 (Scc1), and stromalin 1 (STAG1) or stromalin 2 (STAG2). Cohesin holds sister chromatid pairs together from DNA replication until mitosis (Uhlmann and Nasmyth, 1998; Losada et al., 1998; Murayama et al., 2018; Nasmyth and Haering, 2005). The biorientation of sisters at metaphase requires cohesion, which can be established between sister chromatids during DNA replication and in G2 following a double-strand break (DSB) (Uhlmann and Nasmyth, 1998; Ström and Sjögren, 2005). Cohesin enforces DSB repair from the sister template. Other SMC protein complexes include condensin and SMC5/6. SMC5/6 plays roles in DNA repair, whereas condensin is crucial for condensation of chromosomes (Aragón, 2018; Thadani and Uhlmann, 2015). A complete picture of how the genome is transmitted from the germline into the next generation requires the field to understand how SMC complexes support the maintenance of the genome during this crucial transition.

The molecular events underlying the earliest stages of embryogenesis and how they are linked to the previous meiosis are foundational to development. Relatively few maternal-effect genes have been demonstrated to be essential to support blastocyst development in mouse oocytes, although more are suspected to exist (Kim and Lee, 2014; Condic, 2016). Knowledge of the working mechanisms of maternal-effect genes is essential to understand developmental competence. Most maternal-effect genes have been shown to support imprinting and zygotic genome activation. The RAD21 subunit of cohesin and the cohesin-associated DNA-binding protein CCCTC-binding factor (CTCF) have both been shown to act as maternal-effect genes, with crucial roles in reprogramming and gene expression in the embryo (Ladstätter and Tachibana-Konwalski, 2016; Fedoriw et al., 2004; Wan et al., 2008). *Smc5* depletion in oocytes with the *Zp3-cre* driver leads to segregation-incompetent bivalents during meiosis I (Hwang et al., 2017). Depletion of cohesin subunits in oocytes may leave cohesin associated with chromosomes because sister chromatid cohesion is established during premeiotic S phase and cohesin does not turn over, although age-associated erosion can lead to aneuploidy (Burkhardt et al., 2016; Tachibana-Konwalski et al., 2010; Hodges et al., 2005). Furthermore, the dosage of cohesin can affect chromosome structure and behavior in mouse oocytes (Murdoch et al., 2013). Among the four modular subunits, SMC3 is the only subunit that exists in all cohesin complexes; thus, its ablation will affect all mitotic and meiotic complexes. Our previous study indicated that depletion of histone deacetylase 8 (*Hdac8*), a key recycling factor for SMC3, in mouse oocytes does not block embryogenesis (Singh et al., 2019). However, how *Smc3* contributes to genome integrity and transmission in mouse oocytes and zygotes has not been examined.

We demonstrate herein that *Smc3* in oocytes is required for female fertility and integrity of the zygotic genome. We report that depletion of SMC3 in mouse oocytes causes infertility as a result of failed embryogenesis. Unlike most maternal-effect genes characterized to date, *Smc3* is required to maintain the integrity and segregation of zygotic chromosomes. Depletion of SMC3 in oocytes leads to a series of molecular events that include: (1) persistence of DNA damage following DNA replication; (2) cohesion defects and lagging chromosomes at the first cleavage; (3) sister chromatid missegregation with elongated spindles; (4) micronuclei; and (5) arrest at the two-cell stage. Remarkably, and in contrast to adult females, the dosage of maternal SMC3 in juvenile mutant females is sufficient to support developmental competence, despite compromised centromeric cohesion in the zygote. We propose that maternal SMC3 in the oocyte is required to support the integrity and transmission of the zygotic genome following DNA replication. Despite the high rates of aneuploidy observed during early embryogenesis, these results highlight the fundamental importance of euploidy at the two-cell stage. Furthermore, our study suggests that zygotes can bypass chromosome cohesion defects and chromosome missegregation at the first cleavage, but that aneuploidy may be a deal breaker for the progression of two-cell stage embryos.

## RESULTS

### Conditional deletion of maternal *Smc3* results in female infertility

To study the role of *Smc3* in female germ cells, we used a conditional gene knockout strategy (cKO) based on two Cre drivers. *Gdf9-iCre* and *Zp3-Cre* each express Cre recombinase in oocytes at different stages of development (Lan et al., 2004). Whereas *Zp3-Cre* expression in oocytes by postnatal day (P) 3 is uncontested, the expression of *Gdf9-iCre* at 13.5 days post conception (dpc) has been debated (Ladstätter and Tachibana-Konwalski, 2016; Lan et al., 2004). To detect the timing of *Gdf9-iCre* expression, we examined female gonads with a *lacZ* reporter. We observed β-galactosidase staining in female gonads at 13.5 dpc (Fig. S1), suggesting that *Gdf9-iCre* deletes the floxed-*Smc3* allele in oocytes during premeiotic DNA replication. Next, we validated the efficiency of the Cre recombinases in western blots, detecting SMC3 levels in *Smc3^fl/fl^;Gdf9-iCre* and *Smc3^fl/fl^;Zp3-Cre* oocytes along with wild-type (*Smc3^fl/fl^*) controls. The level of SMC3 significantly decreased by 54% and 77% in *Smc3^fl/fl^;Gdf9-iCre* and *Smc3^fl/fl^;Zp3-Cre* germinal vesicle (GV)-stage oocytes, respectively, compared with controls (Fig. S2A-C), indicating that the cKO strategy was successful, but that SMC3 persists after gene deletion.

**Fig. 1.**
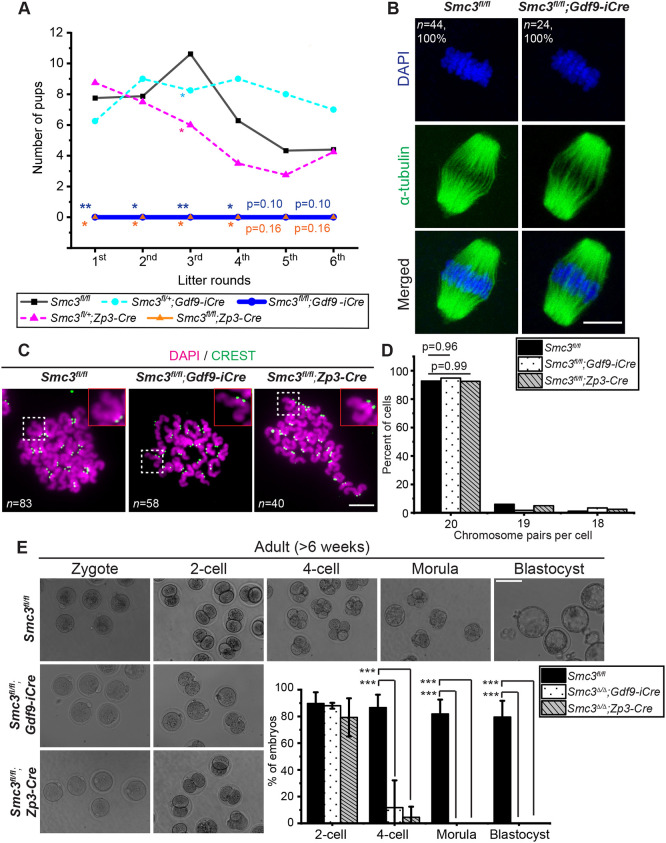
**Maternal *Smc3* is essential for embryogenesis but not meiosis.** (A) Conditional deletion of *Smc3* in mouse oocytes using *Gdf9-iCre* and *Zp3-Cre* and its effect on female fertility. Each female was crossed to produce multiple litters to examine the effect of age on fertility. *n*=3-8 mice for each genotype. The number of pups from each breeder is shown in Table 1. Mann–Whitney *U*-tests were performed to determine the statistical significance between genotypes compared with the wild type (*Smc3^fl/fl^*) at each litter round. Data are means. *P<0.05; **P<0.01. (B) Metaphase II oocytes from adult *Smc3^fl/fl^* and *Smc3^fl/fl^;Gdf9-iCre* female mice fixed and stained with DAPI and α-tubulin antibody to examine chromosome and spindle fiber structures. Although many oocytes were collected from multiple animals (*n*=44 oocytes from six animals for *Smc3^fl/fl^* and *n*=24 oocytes from five animals for *Smc3^fl/fl^;Gdf9-iCre*), we assessed only cases with the spindle in a favorable orientation. (C) Chromosome spreads were performed from metaphase II oocytes from female mice of the indicated genotype. Chromosomes were stained with DAPI and kinetochore antibody (CREST) to score the chromosome number. *n=*6 animals for *Smc3^fl/fl^*, 6 for *Smc3^fl/fl^;Gdf9-iCre* and 3 for *Smc3^fl/fl^;Zp3-Cre*. The magnified images indicate an individual pair of sister chromatids in the dashed squares. (D) Quantification of the number of chromosome pairs per cell from C. Chi-square tests were performed to determine the statistical significance between genotype groups. (E) Zygotes from *Smc3^fl/fl^*, *Smc3^fl/fl^;Gdf9-iCre* and *Smc3^fl/fl^;Zp3-Cre* adult female mice were isolated and cultured *in vitro* to score early embryogenesis. The percentage of embryos that progressed to each stage is shown in the lower right panel. *n*=3-8 repeats from each genotype. The total number of scored embryos is shown in Table 2. Data are mean±s.d. An unpaired, two-tailed Student's *t*-test was used to determine statistically significant differences between genotypes. ***P<0.001. Scale bars: 20 µm in B,C; 100 µm in E.

We next investigated whether deletion of *Smc3* affects female fertility by carrying out a breeding trial. *Smc3^fl/fl^;Gdf9-iCre* and *Smc3^fl/fl^;Zp3-Cre* adult female mice were both sterile (Fig. 1A, Table 1). Although there was variation among individuals, the heterozygous KO *Smc3^+/fl^ Zp3-Cre* female mice remained fertile. This indicates that a single copy of *Smc3* is sufficient to maintain developmentally competent oocytes. The level of SMC3 in heterozygotes was comparable to cKO zygotes and 66% lower than in controls (Fig. S2C). However, protein levels measured by western blot may be an imperfect indicator of function. Overall, we conclude that maternal *Smc3* is required in mouse oocytes for female fertility.

**Table 1.**
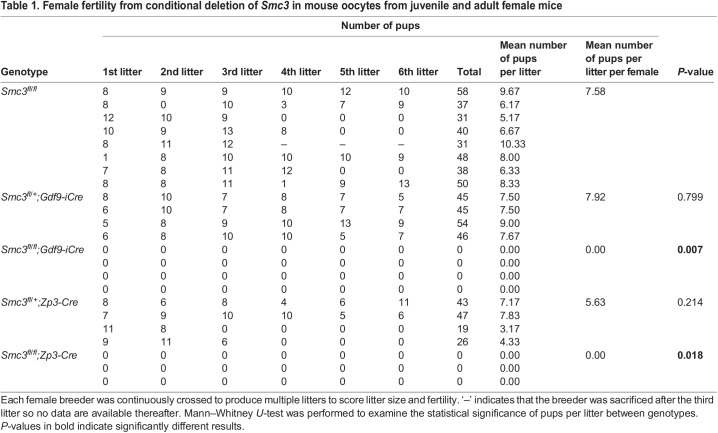
Female fertility from conditional deletion of *Smc3* in mouse oocytes from juvenile and adult female mice

**Table 2.**
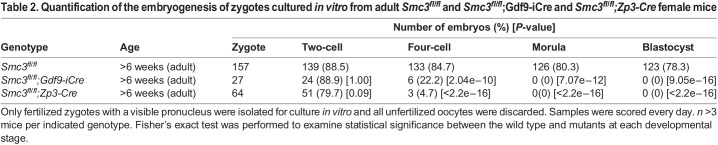
Quantification of the embryogenesis of zygotes cultured *in vitro* from adult *Smc3^fl/fl^* and *Smc3^fl/fl^*;Gdf9-iCre and *Smc3^fl/fl^;Zp3-Cre* female mice

### Maternal *Smc3* is essential for embryogenesis

We next examined how deletion of *Smc3* in oocytes blocks reproduction. Based on female infertility with both *Gdf9-iCre* and *Zp3-Cre* drivers and by scoring the number of follicles in ovaries, we investigated whether loss of *Smc3* impacts ovarian reserve. We performed Hematoxylin and Eosin (H&E) staining on tissue sections of ovaries from adult female mice without hormone stimulation. Corpus lutea were observed in *Smc3^fl/fl^*, *Smc3^fl/fl^;Gdf9-iCre* and *Smc3^fl/fl^;Zp3-Cre* female mice, suggesting that these animals ovulate, and ruling out lack of ovulation as a reason for sterility (Fig. S3A). Furthermore, the total number of follicles was comparable in adult *Smc3^fl/fl^*, *Smc3^fl/fl^;Gdf9-iCre* and *Smc3^fl/fl^;Zp3-Cre* female mice (Fig. S3A,B), suggesting that differences in ovarian reserve do not account for infertility. Furthermore, ovaries from *Smc3^fl/fl^;Gdf9-iCre* and *Smc3^fl/fl^;Zp3-Cre* female mice contained a higher ratio of secondary and antral follicles (Fig. S3C), suggesting that follicle maturation is not compromised but instead appears to be accelerated. Although we cannot explain why loss of *Smc3* would accelerate follicle maturation, our results suggest that loss of *Smc3* does not compromise fertility via depletion of the ovarian reserve. Next, we examined whether maternal *Smc3* in oocytes was required for meiosis. Both *Smc3^fl/fl^;Gdf9-iCre* and *Smc3^fl/fl^;Zp3-Cre* metaphase II oocytes had visibly normal meiotic spindles (Fig. 1B). We further asked whether loss of *Smc3* caused aneuploidy in oocytes. To measure ploidy, we performed chromosome spreads in metaphase I and metaphase II oocytes. There were no obvious defects in chromosome structure in *Smc3^fl/fl^;Gdf9-iCre* and *Smc3^fl/fl^;Zp3-Cre* oocytes (Fig. 1C; Fig. S4). Furthermore, we observed a normal number of chromosome pairs in *Smc3^fl/fl^;Gdf9-iCre* and *Smc3^fl/fl^;Zp3-Cre* metaphase II oocytes (Fig. 1D). Despite the differences in timing of the cre drivers, the levels of SMC3 in the oocytes appeared to be sufficient for chromosome segregation during meiosis in both cases.

Next, we asked whether loss of *Smc3* impacts embryogenesis. Zygotes derived from *Smc3^fl/fl^*, *Smc3^fl/fl^;Gdf9-iCre* and *Smc3^fl/fl^;Zp3-Cre* female mice crossed with wild-type males were isolated and cultured *in vitro*. Given that the phenotype of zygotes is dominated by proteins inherited from the oocyte, and transcription is negligible prior to the first mitotic division (Hamatani et al., 2004; Aoki et al., 1997), we designated the genotype of the zygote according to the maternal allele. Zygotes from *Smc3^fl/fl^;Gdf9-iCre* and *Smc3^fl/fl^;Zp3-Cre* adult female mice arrested predominantly at the two-cell stage (Fig. 1E, Table 2), whereas *Smc3^fl/fl^;Gdf9-iCre* and *Smc3^fl/fl^;Zp3-Cre* embryos failed to mature to morula and blastocyst. Given that the *Smc3^fl/fl^;Gdf9-iCre* and *Smc3^fl/fl^;Zp3-Cre* zygotes shared the same arrest phenotype, together they provide experimental replication. Moving forward, we focused on zygotes from the *Smc3^fl/fl^;Zp3-Cre* driver because the *Zp3-Cre* driver is the standard driver used to study maternal-effect genes. Hereafter, for simplicity, we refer to *Smc3^fl/fl^;Zp3-Cre* zygotes as *Smc3*^Δ/Δ^ zygotes, recognizing that gene deletion leads to protein depletion. Our findings strongly suggest that depletion of maternal SMC3 in oocytes causes infertility by impacting embryogenesis rather than oogenesis.

### Spontaneous DNA damage following DNA replication persists in *Smc3*^Δ/Δ^ cKO zygotes

**Fig. 2.**
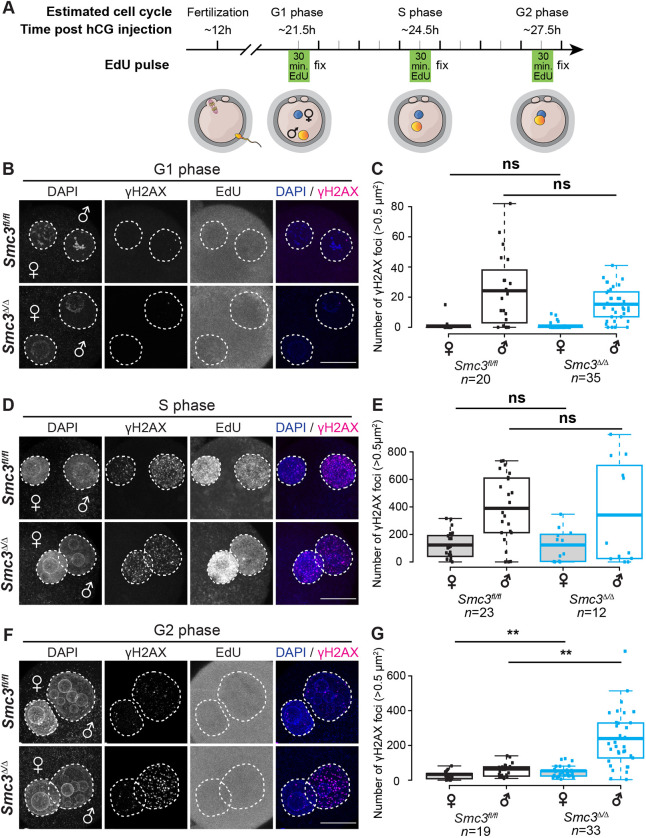
**Spontaneous DNA lesions are elevated in *Smc3*^Δ/Δ^ cKO zygotes following DNA replication.** (A) Cartoon explaining the experimental design and staging. Zygotes from adult *Smc3^fl/fl^* and *Smc3*^Δ/Δ^ cKO female mice were isolated, cultured *in vitro* and fixed at the indicated time point. Cell cycle stage was determined by incubation with EdU 30 min prior to fixation. Zygotes were scored using the Click-iT assay and labeled with γH2AX (magenta) and DAPI (blue) in B-G. (B) Immunostaining of *Smc3^fl/fl^* and *Smc3*^Δ/Δ^ cKO G1-phase zygotes. (C) Quantification of γH2AX foci in *Smc3^fl/fl^* and *Smc3*^Δ/Δ^ cKO G1-phase zygotes. (D) Immunostaining of S-phase zygotes from adult *Smc3^fl/fl^* and *Smc3*^Δ/Δ^ cKO female mice. (E) Quantification of γH2AX foci in *Smc3^fl/fl^* and *Smc3*^Δ/Δ^ cKO S-phase zygotes. (F) Immunostaining of *Smc3^fl/fl^* and *Smc3*^Δ/Δ^ cKO G2-phase zygotes. (G) Quantification of γH2AX foci in *Smc3^fl/fl^* and *Smc3*^Δ/Δ^ cKO G2-phase zygotes. Dashed circles in B,D,E indicate the paternal and maternal pronuclei. Box plots in C,E,G show means (center line); the box limits indicate the range of 1st/3rd quartile and whiskers extend 1.5 times the interquartile range from the 1st and 3rd quartiles. Mann–Whitney *U*-tests were performed to determine the statistical significance between genotypes. **P<0.01. Scale bars: 20 µm.

To examine in more detail how depletion of maternal SMC3 affects embryogenesis, we monitored DNA damage during different phases of the zygote cell cycle (Fig. 2A). *Zp3-cre*-mediated deletion of *Rad21*, a somatic-specific cohesin subunit, leads to unrepaired paternal DNA lesions derived from paternal reprogramming in G1-phase zygotes (Ladstätter and Tachibana-Konwalski, 2016), preventing examination of DNA damage and chromosome segregation at later stages of embryogenesis. Thus, DNA lesions were quantified based on γH2AX foci. In contrast to RAD21 depletion, DNA lesions were similar in *Smc3^fl/fl^* and *Smc3*^Δ/Δ^ G1-phase zygotes (Fig. 2B,C). The SMC3 subunit can be recycled via an acetylation/deacetylation mechanism from one cell cycle to the next (Deardorff et al., 2012; Xiong et al., 2010; Borges et al., 2010; Beckouët et al., 2010). Our results suggest that the recycling of SMC3 is sufficient for the repair of DNA lesions associated with paternal genome reprogramming in *Smc3*^Δ/Δ^ G1-phase zygotes.

DNA replication generates DNA lesions, such as DSBs (Arnaudeau et al., 2001). Spontaneous DNA lesions were observed during S phase in both wild-type and mutant zygotes (Fig. 2D,E), as expected. However, whereas DNA lesions following S phase were detected at low levels in *Smc3^fl/fl^* zygotes, indicating repair, these lesions persisted in *Smc3*^Δ/Δ^ G2-phase zygotes (Fig. 2F,G). Given that we did not induce exogenous DNA damage, our findings strongly suggest that DNA lesions were produced spontaneously during S phase. Therefore, we speculate that maternal *Smc3* is required for the repair of spontaneous DNA lesions that arise during DNA replication, as postulated based on work in budding yeast (Ström and Sjögren, 2005).

**Fig. 3.**
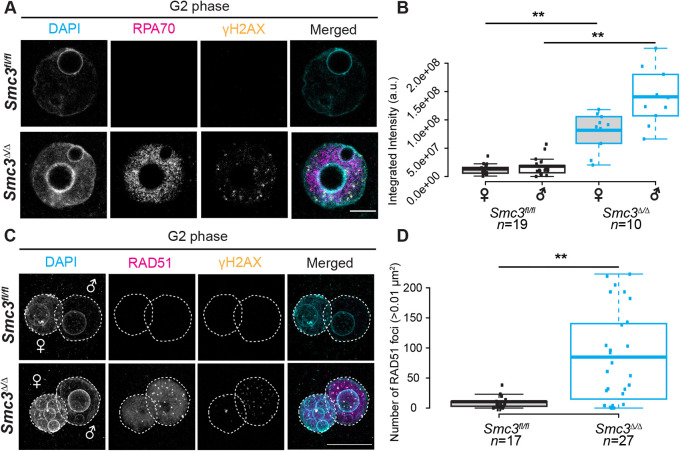
**Loss of maternal *Smc3* is associated with persistent DNA lesions in zygotes.** G2-phase zygotes were isolated, cultured *in vitro*, incubated with EdU and fixed as described in Fig. 2. (A) Immunostaining of G2-phase zygotes from adult *Smc3^fl/fl^* and *Smc3*^Δ/Δ^ cKO female mice for RPA70 (magenta), γH2AX (yellow) and DAPI (cyan). (B) Quantification of the integrated intensity of RPA70 in *Smc3^fl/fl^* and *Smc3*^Δ/Δ^ cKO G2-phase zygotes. (C) Immunostaining of G2-phase zygotes from adult *Smc3^fl/fl^* and *Smc3*^Δ/Δ^ cKO female mice for RAD51 (magenta), γH2AX (yellow), and DAPI (cyan). Dashed circles indicate the paternal and maternal pronuclei. (D) Quantification of RAD51 foci in *Smc3^fl/fl^* and *Smc3*^Δ/Δ^ cKO G2-phase zygotes. Box plots show means (center line), with box limits and whiskers as in Fig. 2G. Mann–Whitney *U*-tests were performed to examine statistical significance between genotypes. **P<0.01. Scale bar: 10 µm in A; 20 µm in C.

To examine further the persistent DNA lesions in G2-phase zygotes, we probed for single-stranded DNA (ssDNA), which is generated during DNA replication. We found that RPA70, a marker of ssDNA, was significantly elevated in *Smc3*^Δ/Δ^ G2-phase zygotes (Fig. 3A,B). Although ssDNA can be generated from either active transcription or replication, transcription is negligible in mouse zygotes (Aoki et al., 1997; Jukam et al., 2017). Therefore, ssDNA is predicted to derive primarily from DNA replication. Our data suggest that maternal *Smc3* is required to complete repair events associated with DNA replication in zygotes.

Homologous recombination is a common method used to restart stalled replication forks (Arnaudeau et al., 2001). We investigated whether RAD51 persists into G2 phase when SMC3 is depleted. We observed a significantly higher incidence of RAD51 foci in *Smc3*^Δ/Δ^ zygotes compared with *Smc3^fl/fl^* (Fig. 3C,D), consistent with the γH2AX data, and demonstrating that accumulation of DNA lesions in *Smc3*^Δ/Δ^ zygotes coincides with the accumulation of homologous recombination proteins. Thus, our findings suggest that maternal SMC3 is necessary to complete the repair of spontaneous DNA damage from S phase in zygotes but is not essential for the recruitment of homologous recombination machinery.

### Deletion of maternal *Smc3* leads to lagging chromosomes, loss of cohesion and micronuclei during the first mitotic division

The first detectable phenotype following loss of *Smc3* in oocytes was persistent DNA damage in G2-phase zygotes. However, zygotes completed the first mitotic division. We next asked why *Smc3*^Δ/Δ^ embryos arrest at the two-cell stage. Although the two-cell stage is a common arrest point in mouse embryo development, the checkpoints involved are not well characterized (Zanoni et al., 2009). Given that zygotic genome activation (ZGA) occurs late in the two-cell stage in mice, faulty ZGA can lead to arrest (Schultz, 2002, 1993). In addition, because cohesin may contribute to ZGA in zebrafish (Meier et al., 2018), we examined whether loss of maternal *Smc3* is required for ZGA in mice. We measured global RNA transcription in *Smc3*^Δ/Δ^ embryos at the two-cell stage using a 5-ethynyl uridine (5-EU) click-iT assay. In this assay, G2-phase zygotes and two-cell stage embryos were pulse labeled with 5-EU, which incorporates into nascent RNA and can be quantified via fluorescence (see Materials and Methods). The signal was comparable between *Smc3^fl/fl^* and *Smc3*^Δ/Δ^ two-cell stage embryos (Fig. S5). Although we did not determine whether the zygotic transcriptome was affected, ZGA-associated nascent transcription occurred with normal timing and to grossly normal levels in *Smc3*^Δ/Δ^ embryos, suggesting that ZGA can initiate.

**Fig. 4.**
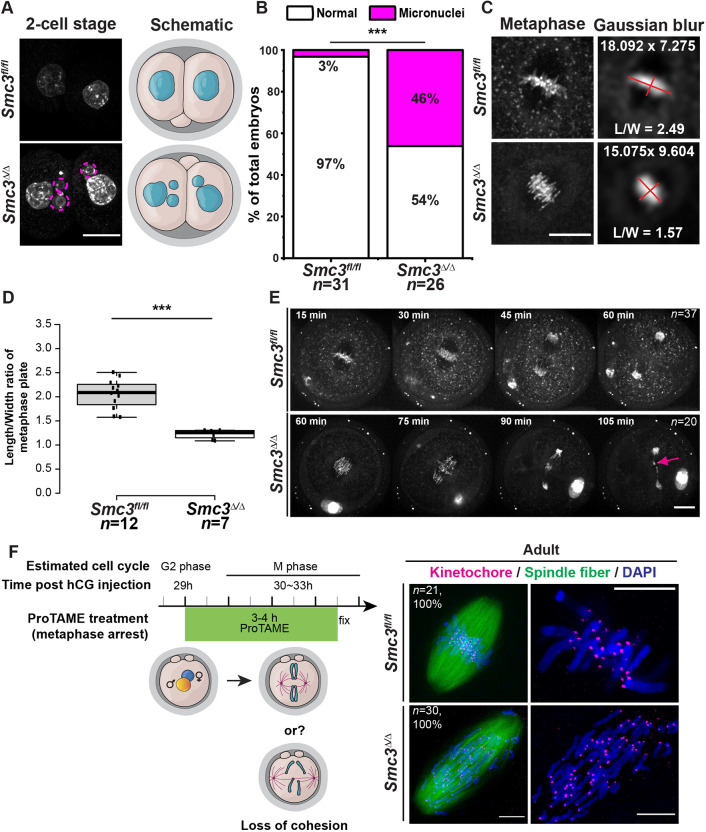
**Loss of maternal *Smc3* is associated with micronuclei and lagging chromosomes during the first mitotic division.** (A) DAPI staining for two-cell stage embryos from adult *Smc3^fl/fl^* and *Smc3*^Δ/Δ^ cKO female mice. Dashed circles indicate micronuclei. A schematic is presented for each genotype. (B) Quantification of micronuclei-positive two-cell-stage embryos from adult *Smc3^fl/fl^* and *Smc3*^Δ/Δ^ cKO female mice. A Chi-square test was performed to determine statistical significance between genotypes. ***P<0.001. (C-E) Live cell imaging of SIR-DNA dye-stained zygotes revealed that (C,D) chromosome morphologies were different and (E) micronuclei were derived from the lagging chromosome during the first mitotic division in *Smc3*^Δ/Δ^ cKO zygotes. (C) *Z* projection of spindle chromosomes was Gaussian blurred and the length and width (red lines) were measured. (D) Quantification of L/W ratio of spindle chromosomes in *Smc3^fl/fl^* and *Smc3*^Δ/Δ^ metaphase zygotes. Only embryos with correct orientation were counted. Box plots show means (center line), with box limits and whiskers as in Fig. 2G. An unpaired, two-tailed Student's *t*-test was performed to examine statistical significance between genotypes. ***P<0.001. (E) Time-lapse montages of *Smc3^fl/fl^* and *Smc3*^Δ/Δ^ zygotes. The magenta arrow indicates the micronucleus formed from lagging chromosomes. See Movies 1 and 2 for the entire process at high resolution. (F) Immunostaining of metaphase zygotes from adult *Smc3^fl/fl^* and *Smc3*^Δ/Δ^ cKO animals for kinetochores (CREST), spindle fibers (α-tubulin), and chromosomes (DAPI). *In vitro*-cultured zygotes were incubated with ProTAME to synchronize/arrest at metaphase (*n*=21 *Smc3^fl/fl^*; 30 *Smc3*^Δ/Δ^). Magnified images of kinetochore and chromosomes are presented in the right panel. Scale bars: 10 µm in A,C,F; 20 µm in E.

Micronuclei are a hallmark of genome instability (Soto et al., 2018). They often form when a chromosome or fragment fails to incorporate into the daughter nucleus during cell division. We asked whether depletion of maternal SMC3 results in micronuclei during the first zygotic division, detected by DNA staining in two-cell stage embryos. Remarkably, we found that *Smc3*^Δ/Δ^ embryos had significantly more micronuclei compared with *Smc3^fl/fl^* embryos (Fig. 4A,B). This strongly suggests that loss of maternal *Smc3* in the oocyte results in chromosome instability during the first mitotic division. To examine the formation of micronuclei in more detail, we used live cell imaging. DNA in zygotes was stained with the low cell-toxicity SIR-DNA dye and imaged every 3 min by spinning disk confocal microscopy. This allowed us to visualize the entire process of chromosome segregation during the first mitotic division in unperturbed zygotes [Movie 1 (wild type), Movie 2 (*Smc3*^Δ/Δ^), Fig. 4C-E]. The morphology of sister chromatids in *Smc3*^Δ/Δ^ zygotes during metaphase appeared more stretched, consistent with reduced centromeric cohesion (Fig. 4C,D). Moreover, we observed lagging chromosomes during anaphase that formed a micronucleus (Fig. 4E). In all 20 movies of *Smc3*^Δ/Δ^ embryos, lagging chromosomes were observed, as compared to one in 37 movies of *Smc3^fl/fl^* embryos. We speculate that weakened centromeric cohesion results in merotelic kinetochore attachments, with kinetochores attached to microtubules from both spindle poles, a configuration that evades detection by the spindle assembly checkpoint, culminating in lagging chromosomes that form micronuclei (Haarhuis et al., 2013; Thompson and Compton, 2011). Our results demonstrate that maternal *Smc3* is required for accurate chromosome segregation during the first mitotic division. Importantly, chromosome missegregation and aneuploidy preceded ZGA and the two-cell arrest.

Cohesin is well known for its role in sister chromatid cohesion, which facilitates biorientation and chromosome segregation. Given that our live cell imaging revealed lagging chromosomes in *Smc3*^Δ/Δ^ zygotes, we next asked whether loss of maternal *Smc3* affects sister chromatid cohesion. To achieve this, we induced metaphase arrest with the pharmacological anaphase-promoting complex/cyclosome (APC/C) inhibitor proTAME and then examined centromeres and spindles. We observed a complete loss of sister chromatid cohesion and separation in all *Smc3*^Δ/Δ^ zygotes (*n*=21), and maintenance in all *Smc3^fl/fl^* zygotes (*n*=30) (Fig. 4F). Given that precocious separation of whole chromosomes was not apparent in live cell imaging of unperturbed mutant zygotes, we suggest that proTAME exacerbates the weakened centromeric cohesion observed in live cell imaging because of prolonged metaphase arrest, with intact microtubules generating cohesion fatigue and precocious sister separation in *Smc3*^Δ/Δ^ zygotes. We conclude that maternal SMC3 is required in zygotes to create robust sister chromatid cohesion and prevent lagging chromosomes, micronuclei formation and aneuploidy.

We investigated whether a DNA-damage checkpoint is activated in *Smc3*^Δ/Δ^ embryos at the two-cell stage. To examine the canonical ATR and ATM checkpoints at this stage, we labeled Chk1 and phospho-ATM (p-ATM), respectively (Fig. S6). We first validated the antibodies by introducing replication stress using hydroxyurea (HU) treatment in wild-type G2-phase zygotes. We found that Chk1 increased in G2-phase zygotes, whereas p-ATM remained low (Fig. S6A,B). Although we did not detect p-ATM signal in G2-phase zygotes with HU treatment, this antibody was validated in our lab in mouse placental tissue in a recent study (Singh et al., 2020). We next examined Chk1 and p-ATM levels in two-cell stage embryos. Neither Chk1 nor p-ATM increased at this stage in *Smc3*^Δ/Δ^ embryos, suggesting that the canonical ATM and ATR checkpoints were not activated (Fig. S6C,D). However, checkpoints in early embryogenesis are weak or may be non-canonical (Conn et al., 2004; Vázquez-Diez et al., 2019; Adiga et al., 2007; Kato and Tsunoda, 1992). Nonetheless, aneuploidy at the two-cell stage is not tolerated because embryos do not progress to further developmental stages (Pauerova et al., 2020), suggesting that aneuploidy (i.e. micronuclei) underpins the two-cell arrest in *Smc3*^Δ/Δ^ embryos.

### Developmental competence remains intact in mutant juveniles

**Fig. 5.**
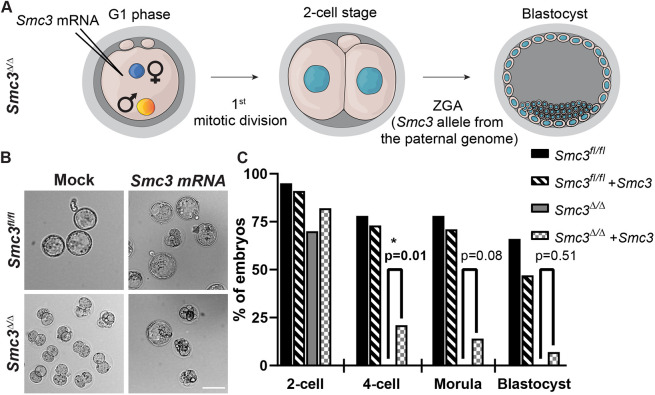
**Developmental competence of the *Smc3*^Δ/Δ^ cKO zygote is rescued by microinjection of *Smc3*.** (A) Cartoon explaining the experimental design and staging. *Smc3* mRNA or TE buffer (mock) was microinjected into the *Smc3*^Δ/Δ^ cKO zygote in G1 phase. If the mutant zygotes successfully completed the first mitotic division, *Smc3* expressed from the paternal genome should enable embryogenesis after ZGA. (B) Microinjected embryos were cultured *in vitro*. (C) Quantification of early embryogenesis as a percentage of zygotes that successfully progressed to the stage indicated. The total number of embryos at each stage is shown in Table 3. Fisher's exact test was performed to examine the statistical significance between indicated genotypes at each stage. *P<0.05. Scale bar: 100 µm in B.

If persistent DNA damage and aneuploidy underpin the two-cell arrest, then rescuing this single round of DNA repair and chromosome segregation may permit embryogenesis, once *Smc3* can be expressed from the paternal genome during ZGA. To determine whether rescue could be achieved, we delivered exogeneous *Smc3* mRNA into *Smc3*^Δ/Δ^ zygotes using microinjection (Fig. 5A,B) prior to S phase. Whereas all mock-injected *Smc3*^Δ/Δ^ zygotes arrested at either the zygote or two-cell stage (31 total), ∼22% of microinjected mutant zygotes (nine of 44 embryos) continued past the two-cell stage, six embryos successfully matured to a morula and two matured to a blastocyst (Fig. 5C, Table 3). Therefore, our data suggest that increased levels of SMC3 in zygotes enable the first mitosis and rescue embryogenesis. Microinjection is a challenging method; the imperfect rescue could arise from several factors, including: (1) heterogeneity in the timing of mating among different female mice, such that a few zygotes enter S phase prior to microinjection, although the time was selected based on staging from Fig. 2; or (2) insufficient translation of SMC3 in some zygotes. However, the rescue of embryogenesis in a subset of zygotes is consistent with the idea that sufficient SMC3 provided during the correct time window can enable developmental competence.

**Table 3.**
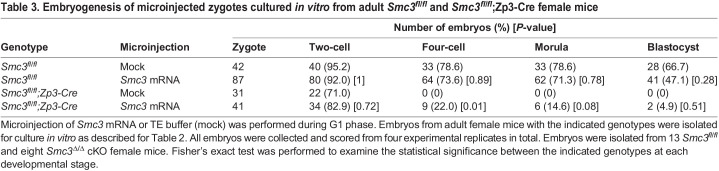
Embryogenesis of microinjected zygotes cultured *in vitro* from adult *Smc3^fl/fl^* and *Smc3*^*fl/fl*^;Zp3-Cre female mice

**Fig. 6.**
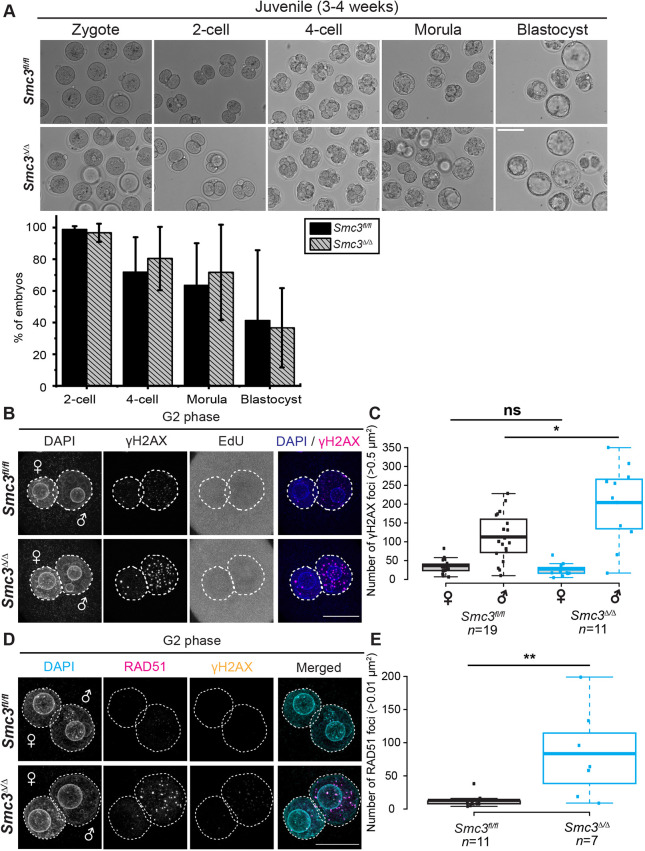
**Developmental competence of the *Smc3*^Δ/Δ^ cKO zygote is enabled in juvenile females (3-4 weeks old).** (A) Embryos from juvenile females of the indicated genotype were cultured *in vitro* for the indicated length of time following the conditions in Fig. 1E. The quantification of early embryogenesis is shown in the lower histogram. *n*=3 repeats from each genotype. The total number of embryos scored at each stage is shown in Table 4. Data are mean±s.d. An unpaired, two-tailed Student's *t*-test showed no statistically significant differences between genotypes. (B) Zygotes in G2 phase, derived from juvenile females with the indicated genotype, were immunostained for γH2AX as described in Fig. 2. (C) Quantification of γH2AX foci from the conditions in B. Mann–Whitney *U*-tests were performed to determine the statistical significance between genotypes. *P<0.05. (D) G2-phase zygotes from juvenile females of the indicated genotype were immunostained for RAD51 (magenta), γH2AX (yellow) and DAPI (cyan). (E) Quantification of RAD51 foci in juvenile *Smc3^fl/fl^* and *Smc3*^Δ/Δ^ cKO G2-phase zygotes from D. Dashed circles in B,D indicate the paternal and maternal pronuclei. Box plots are means (center line), with box limits and whiskers as in Fig. 2G. Mann–Whitney *U*-tests were performed to determine the statistical significance between genotypes. **P<0.01. Scale bars: 20 µm in B,D; 100 µm in A.

In addition to microinjection, we asked whether endogenous SMC3 protein could rescue the two-cell arrest. We hypothesized that SMC3 levels in oocytes may decline starting at P3 with induction of the *Zp3-Cre* driver and continue until sexual maturity, leading to a gradual loss of developmental competence. If true, juvenile females might have more SMC3, which would enable the first mitotic division and developmental competence. We asked whether oocytes from juvenile *Smc3*^Δ/Δ^ female mice were developmentally competent. Given that most mouse strains reach sexual maturity at 4-7 weeks of age, we could not conduct a breeding trial. Instead, 3- to 4-week-old juvenile *Smc3*^Δ/Δ^ female mice were treated with hormones for superovulation, followed by mating, zygote collection and culture *in vitro*. Remarkably, zygotes from juveniles matured to blastocysts (Fig. 6A, Table 4). Also, the level of SMC3 in juvenile *Smc3*^Δ/Δ^ GV-stage oocytes was comparable to *Smc3^fl/fl^* levels by western blot analysis (Fig. S7A,B). Therefore, our data strongly suggest that the dosage of maternal SMC3 in oocytes declines between the juvenile and sexually mature stages in the mutant. Increased levels of SMC3 in the juvenile-derived oocytes enable successful chromosome segregation at the first mitosis, such that developmental competence is achieved.

**Table 4.**
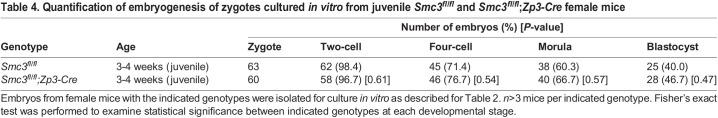
Quantification of embryogenesis of zygotes cultured *in vitro* from juvenile *Smc3^fl/fl^* and *Smc3*^*fl/fl*^;*Zp3-Cre* female mice

We then asked whether levels of SMC3 in juveniles were sufficient for repair of DNA lesions in *Smc3*^Δ/Δ^ zygotes. We quantified γH2AX foci in juvenile-derived *Smc3^fl/fl^* and *Smc3*^Δ/Δ^ zygotes in G2 phase (Fig. 6B,C). Surprisingly, we found that paternal DNA lesions increased in zygotes derived from *Smc3^fl/fl^* juvenile females compared with mature females, suggesting either a general insufficiency of repair or an elevation in the incidence of lesions. Paternal DNA lesions were further increased in zygotes derived from juvenile *Smc3*^Δ/Δ^ females, whereas maternal DNA lesions showed no difference, suggesting a partial rescue. To examine whether DNA repair machinery was affected in zygotes derived from juvenile *Smc3*^Δ/Δ^ females, we quantified RAD51 foci as described previously (Fig. 6D,E). RAD51 foci were similar in zygotes derived from juvenile and mature *Smc3^fl/fl^* females. However, RAD51 foci were elevated in zygotes derived from juvenile *Smc3*^Δ/Δ^ females compared with *Smc3^fl/fl^* females, similar to the observations in zygotes derived from mature *Smc3*^Δ/Δ^ females. Our findings suggest that zygotes from wild-type juvenile females have persistent DNA lesions compared with zygotes from mature females, complicating the interpretation of the results. However, juvenile-derived mutant oocytes appear to contain sufficient SMC3 to partially prevent persistent DNA damage and enable the first mitotic division in *Smc3*^Δ/Δ^ zygotes. Importantly, once ZGA occurs, *Smc3* provided by the paternal genome appears to be sufficient for developmental competence, once the first zygotic division is successfully completed.

**Fig. 7.**
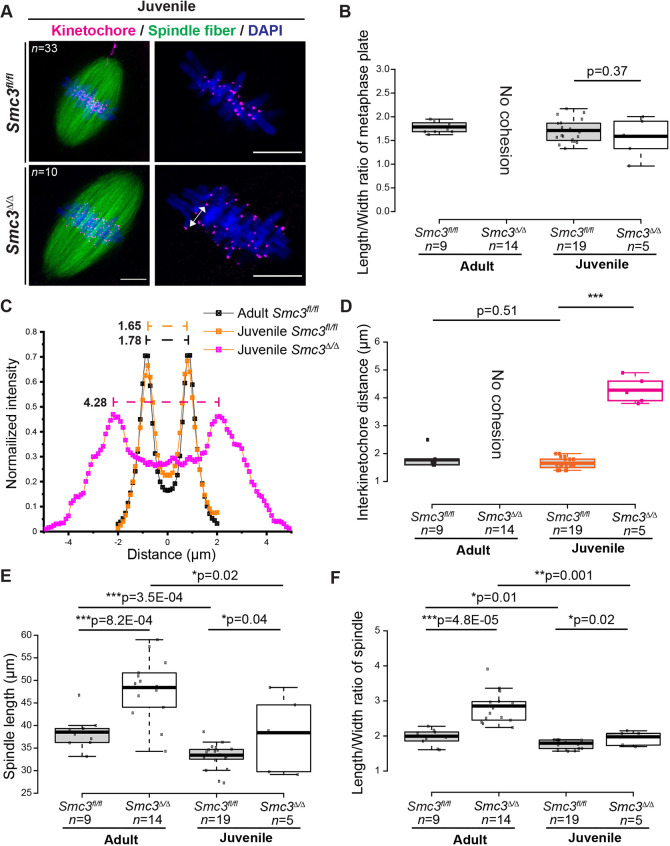
**Loss of maternal *Smc3* disrupts sister chromatid cohesion during the first mitotic division.** Zygotes cultured *in vitro* from adult and juvenile *Smc3^fl/fl^* and *Smc3*^Δ/Δ^ cKO female mice were incubated with an APC inhibitor to arrest at metaphase. (A) Metaphase-arrested and fixed zygotes from juvenile *Smc3^fl/fl^* and *Smc3*^Δ/Δ^ cKO female mice were stained to visualize kinetochores (CREST, magenta), spindle fibers (α-tubulin, green), and DNA (DAPI, blue). Compared with Fig. 4D, the phenotype between adult and juvenile *Smc3*^Δ/Δ^ zygotes is easily distinguished. The double-headed arrow indicates centromeres separated from an individual sister chromatid pair. (B) Quantification of L/W ratio of spindle chromosomes from adult (Fig. 4F) and juvenile (A) *Smc3^fl/fl^* and *Smc3*^Δ/Δ^ metaphase zygotes. Metaphase zygotes were incubated with the APC inhibitor. Only the embryos with correct orientation were included for quantification. (C) The interkinetochore distance was measured by centromere signals between individual sister chromatid pairs. Line profiles of the CREST signal along the *x*-axis were generated from aligning chromosome centers of each chromosome pair shown in A and Fig. 4F. The average profile of all chromosome pairs in individual zygotes was then generated by fitting line profiles to a double Gaussian model**.** The two peaks of CREST signal determined the position of centromere centers. The dashed line indicates the interkinetochore distance of chromosome pairs in indicated genotypes. The sample size is indicated in D. (D) Quantification of the interkinetochore distance in metaphase zygotes from adult and juvenile *Smc3^fl/fl^* and *Smc3*^Δ/Δ^ cKO female mice. The interkinetochore distance was determined as in C. The interkinetochore distance of adult *Smc3*^Δ/Δ^ cKO zygotes could not be quantified because cohesion was completely lost and sister chromatid pairs were indistinguishable. (E,F) Quantifications of length (E) and L/W ratio (F) of spindles from adult and juvenile. Box plots are means (center line), with box limits and whiskers as in Fig. 2G. A one-way ANOVA test with the Tukey HSD test was used to determine statistically significant differences between genotypes in D. Unpaired, two-tailed Student’s *t*-tests were performed to determine the statistical significances between groups in B,E,F. *P<0.05; **P<0.01; ***P<0.001. Scale bars: 10 µm.

Deletion of maternal *Smc3* in oocytes from mature females resulted in the loss of cohesion and sister chromatid separation in zygotes arrested in metaphase (Fig. 4F). We asked whether cohesion was intact in zygotes from juvenile *Smc3*^Δ/Δ^ female mice based on the analysis of spindles and centromeres from metaphase-arrested juvenile-derived *Smc3*^Δ/Δ^ zygotes. Remarkably, chromosomes appeared stretched on the spindle, but precocious separation of entire sister chromatids did not occur (Fig. 7A). The length/width (L/W) ratio of the metaphase plate in juvenile-derived *Smc3*^Δ/Δ^ zygotes was comparable to that in *Smc3^fl/fl^* zygotes (Fig. 7B), suggesting that, unlike in adult-derived mutant zygotes, chromosomes were not completely stretched apart (Fig. 4C,D). Notably, we observed a Y-shaped chromatid structure in spindles from juvenile-derived *Smc3*^Δ/Δ^ metaphase zygotes. We speculate that this Y structure is a consequence of losing centromeric cohesion on telocentric sister chromatids during proTAME arrest, consistent with the idea that prolonged arrest with intact microtubules may challenge centromeric cohesion. We also observed Y-shaped chromatids in chromosome spreads (Fig. S8), indicating that intersister cohesion in the short p-arm of a telocentric chromosome is insufficient to withstand prolonged mitotic arrest. The stretched morphology of sister chromatids on the mitotic spindle of juvenile-derived mutant zygotes in proTAME (Fig. 7A) was reminiscent of the stretched appearance of unperturbed adult-derived mutant zygotes in the live cell imaging experiment (Fig. 4C), suggesting that the stretched appearance represents poor centromeric cohesion.

Interkinetochore distances increase with age in oocytes, indicative of a loss of centromeric cohesion (Duncan et al., 2012), but the physiological relevance of this is not understood. To quantify centromeric cohesion, we measured the interkinetochore distance in zygotes. Intensity profiles of sister kinetochore signals were traced by double Gaussian fitting to determine interkinetochore distances in metaphase (Fig. 7C). We measured all distinguishable chromosome pairs with appropriate orientation in individual zygotes. In adult-derived *Smc3*^Δ/Δ^ metaphase zygotes, we were unable to quantify the interkinetochore distance because sister chromatid cohesion was completely lost and pairs were unidentifiable. In juvenile-derived *Smc3*^Δ/Δ^ metaphase zygotes, the interkinetochore distance was significantly increased relative to *Smc3^fl/fl^* zygotes (Fig. 7C,D). This strongly suggests that juvenile-derived *Smc3*^Δ/Δ^ zygotes exhibit centromeric cohesion defects, but, strikingly, can sustain developmental competence with cohesion in chromosome arms. However, by western blot, we were unable to detect significantly decreased levels of SMC3 in juvenile-derived *Smc3*^Δ/Δ^ oocytes compared with adult-derived *Smc3^fl/fl^* oocytes (Fig. S7A,B). This imperfect correlation between the level of SMC3 and cohesion could be a result of the insensitivity of western blotting, and that protein levels on a western blot are an imperfect indicator of SMC3 actively engaged in cohesion.

Loss of cohesion is associated with increased spindle length in yeast (Stephens et al., 2011), because there is less inward force constraining the elongation (Nannas et al., 2014), although this has not been examined in mouse zygotes. We measured the morphologies of sister chromatids and spindles in proTAME-treated zygotes from both adult and juvenile females (Fig. 7E,F). The L/W ratio and length of the spindle in juvenile-derived *Smc3*^Δ/Δ^ zygotes were significantly lower than in adult-derived *Smc3*^Δ/Δ^ metaphase zygotes, suggesting that the metaphase plate and spindle morphologies improved and cohesion was more substantial (Fig. 7E,F). Moreover, the spindle length and L/W ratio of the spindle in the mutant were significantly higher than in juvenile-derived wild-type zygotes, consistent with the loss of centromeric cohesion. In sum, the improved spindle length, chromosome morphology and DSB repair in juvenile-derived mutant zygotes are consistent with more cohesion than in the adult-derived mutant zygotes. Overall, we conclude that sufficient maternal SMC3 in *Smc3*^Δ/Δ^ juvenile-derived oocytes exists to support chromosome integrity, which enables developmental competence.

## DISCUSSION

Here, we demonstrate that *Smc3* is a maternal-effect gene and that sufficient levels of protein must be present in the oocyte for developmental competence. *Smc3* joins the 60 or so maternal-effect genes that are known to block progression from the two-cell stage (Condic, 2016). SMC3 supports the fundamental processes of chromosome duplication and segregation, distinct from the cohesin subunit RAD21 and the cohesin-binding partner CTCF, which are documented to support gene expression and imprinting (Ladstätter and Tachibana-Konwalski, 2016; Fedoriw et al., 2004; Wan et al., 2008). We demonstrate that maternal SMC3 is required to repair spontaneous DNA lesions following DNA replication in zygotes and to support accurate chromosome segregation during the first mitotic division post fertilization to prevent aneuploidy. Despite the high rates of aneuploidy observed in early embryos in other studies, which suggest that aneuploidy is tolerated, our results suggest that the integrity and transmission of the zygotic genome is essential for successful embryogenesis and rely on proteins stored into the oocyte. Furthermore, we speculate that our findings are relevant to *in vitro* fertilization, in which preimplantation development is often monitored, because our results suggest that elongated spindles in the zygote and micronuclei at the two-cell stage serve as visual indicators of chromosomal instability correlating with poor outcomes.

Depletion of SMC3 in oocytes results in a unique phenotype relative to all other cohesin subunits that have been studied thus far. REC8- and SMC1β-null mutations are sterile because of meiotic failure, whereas *Smc1β^fl/fl^;Gdf9-iCre* female mice are fertile, in contrast to the infertility observed in our breeding trials (Xu et al., 2005; Revenkova et al., 2004, 2010; Bannister et al., 2004). Elimination of meiotic cohesin genes via *Gdf9-iCre* or *Zp3-Cre* drivers may not affect meiotic competence because sufficient protein remains. Given that the zygote starts using mitotic cohesin subunits instead of meiotic subunits after fertilization, elimination of meiotic cohesin may not affect developmental competence. By contrast, elimination of RAD21 in oocytes via *Zp3-Cre* impacts developmental competence via paternal reprogramming and severely compromises entry into mitosis (Ladstätter and Tachibana-Konwalski, 2016). Unlike SMC3, which can be recycled, RAD21 is cleaved and destroyed after each cell division. We speculate that recycling of SMC3 allows cohesin-dependent repair of reprogramming-derived DNA lesions during G1. Alternatively, RAD21 may be uniquely involved in this repair. The first phenotype detected in *Smc3*^Δ/Δ^ zygotes is persistent DNA lesions in G2 phase. However, zygotes efficiently enter mitosis and the majority then arrest at the two-cell stage. We failed to detect canonical ATR and ATM checkpoints in the two-cell-blocked *Smc3*^Δ/Δ^ embryos. Determining the checkpoint mechanism will require further experimentation, but our findings suggest that persistence of DNA damage and loss of cohesion in the zygote do not prompt a checkpoint arrest.

**Fig. 8.**
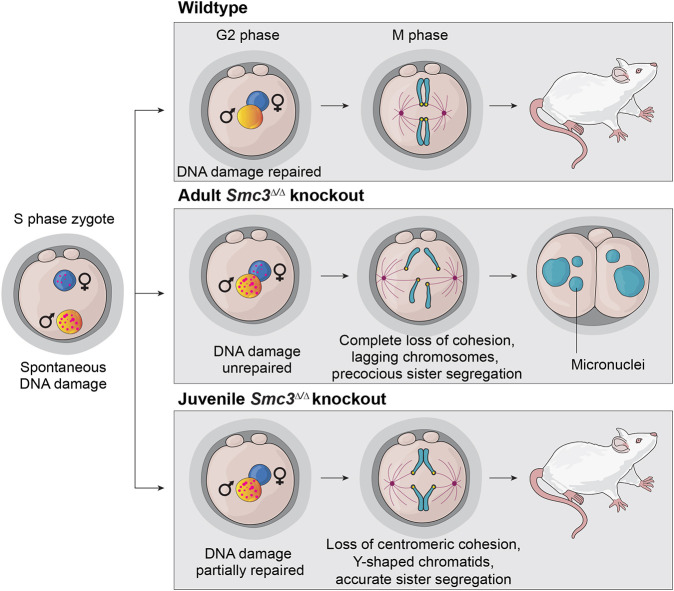
**Schematic summary of results highlighting significant findings.** Spontaneous DNA lesions accumulated following S phase in adult-derived *Smc3*^Δ/Δ^ cKO zygotes, followed by the first mitotic division with lagging chromosomes, micronuclei and arrest at the two-cell stage. Cohesion was undetectable in adult-derived *Smc3*^Δ/Δ^ cKO zygotes. Although juvenile-derived *Smc3*^Δ/Δ^ cKO zygotes lose centromeric cohesion, they can sustain developmental competence with cohesion in the q-arms.

Although many maternal-effect genes point to the vital role of reprogramming and embryonic genome activation, our study suggests a crucial role for chromosome maintenance and euploidy at the two-cell stage, despite many studies demonstrating frequent aneuploidy in early embryos. Studies of the repair of spontaneous DNA damage from DNA replication and sister chromatid cohesion in a single cell cycle have mainly been carried out in mammalian cultured cells and yeast. Zygotes are an elegant model because factors can be depleted in the oocyte prior to the first mitotic division; in addition, zygotes reveal vital functions required in early embryos. Fifty DSBs are estimated to occur during a normal S phase in a human cell (Vilenchik and Knudson, 2003). Our study is consistent with the proposal that cohesin is required to support their repair (Singh et al., 2020; Mondal et al., 2019; Ström and Sjögren, 2005), including replication fork restart (Frattini et al., 2017). Persistence of spontaneous DNA lesions in mutant zygotes is followed by sister chromatid missegregation. Lagging chromosomes and their subsequent incorporation into micronuclei could result from merotelic kinetochore attachment as well as from compromised DNA replication, resulting in tangled chromosomes that lag in segregation and resolve into micronuclei (Fig. 8), generating aneuploidies that are incompatible with developmental progression. *Filia* and *Bcas2* are other maternal-effect genes required to maintain genomic integrity during cleavage-stage mouse embryogenesis, supporting the proposal that euploidy is a prerequisite for developmental competence (Zheng and Dean, 2009; Xu et al., 2015). A recent study found that aneuploidy from two-cell embryos does not propagate into further developmental stages (Pauerova et al., 2020), consistent with our proposal that the mutant embryos arrest at the two-cell stage because of mitosis-derived aneuploidy, although we cannot rule out faulty ZGA.

Centromeric cohesion is considered essential for accurate sister chromatid biorientation and segregation. Our study is consistent with previous studies that show that loss of centromeric cohesion affects the physical architecture of the spindle, resulting in an elongated spindle (Stephens et al., 2011; Deehan Kenney and Heald, 2006). In a surprising twist, the Y-shaped chromatid structure in chromosome spreads derived from juvenile *Smc3*^Δ/Δ^ zygotes, coupled with their ability to progress to the blastocyst stage, suggests that loss of centromeric cohesion can be compatible with successful mitosis and developmental competence. We speculate that the Y-shaped chromatid indicates that cohesion is completely lost surrounding the centromere and throughout the short arm of telocentric chromosomes. Cohesion in the q-arm of sister chromatids in the zygote is then sufficient to enable successful biorientation, mitotic division and subsequent embryogenesis. This observation is paradoxical in light of models based on cultured mammalian cells in which arm cohesion is removed by the prophase pathway and does not contribute to sister chromatid biorientation and segregation (Waizenegger et al., 2000; Sumara et al., 2002; Losada et al., 2002; Warren et al., 2000). Future efforts will be needed to ascertain how and where cohesion is needed for sister chromatid biorientation and segregation in early embryos.

Although the exact mechanism remains unclear, cohesin participates in homologous recombination (Litwin et al., 2018). Lack of cohesin or its loader leads to the accumulation of DNA lesions after γ-irradiation (Ferreira and Cooper, 2004; Sjögren and Nasmyth, 2001; Ström et al., 2007, 2004). However, our RAD51 analysis indicates that depletion of SMC3 does not disable the recruitment of homologous recombination proteins, consistent with a previous study in human cultured cells (Kong et al., 2014). Cohesin surrounds induced DSBs and holds sister chromatids together to facilitate their repair (Litwin et al., 2018; Watrin and Peters, 2006). We speculate that cohesion is established during DNA replication at sites of spontaneous damage and that these normally invisible repair events contribute to the establishment of sister chromatid cohesion during S phase. In this speculative model, first postulated in budding yeast (Ström and Sjögren, 2005), spontaneous DNA damage during DNA replication from stalled forks results in the recruitment of cohesin to support sister chromatid repair. This cohesion then persists and contributes to sister chromatid cohesion for chromosome biorientation during mitosis. However, more work will be required to test this working model.

We demonstrate that genetically identical zygotes can differ in developmental competence depending on the age of the mother. Although SMC3 is considered stable in aging oocytes (Tsutsumi et al., 2014), whether a natural age-associated decline in other cohesin subunits in the oocyte could impact developmental competence is an open question. Our study indicates that fertility is dramatically affected in mutant females over the span of ∼2 weeks, as they progress from a juvenile to an adult. Distinct from studies that demonstrate aging-related cohesion failures in meiosis and infertility (Tsutsumi et al., 2014; Lister et al., 2010; Chiang et al., 2010; Tachibana-Konwalski et al., 2010; Duncan et al., 2012; Hodges et al., 2005; Subramanian and Bickel, 2008; Murdoch et al., 2013), our study suggests that mutant juvenile females achieve developmental competence via higher dosage in oocytes, which benefits zygotes. However, the juvenile stage is also associated with increased DNA lesions and altered spindle morphology in zygotes, even those from wild-type females. Oocyte quality declines with age, but prepubertal females also have poor-quality oocytes (Karavani et al., 2019; Oktay et al., 2021). In the future, careful consideration should be accorded to maternal age when assessing maternal-effect genes because we would have concluded that *Smc3* is not a maternal-effect gene if we had only analyzed zygotes from juveniles, a protocol recommended by some textbooks (Behringer et al., 2014; Luo et al., 2011). Future efforts will benefit from additional depletion strategies, analysis at multiple ages, visualizing cohesin on chromosomes to better appreciate the functional pool, and analysis of additional proteins involved in developmental competence, chromosome segregation and spindle function (Ma et al., 2013; De and Kline, 2013).

In conclusion, our study provides insights into the working mechanism of maternal *Smc3* during early embryonic development. Oocyte-stored SMC3 is required to support the integrity of the zygotic genome during the very first round of DNA replication and sister chromatid segregation to pass successfully through the first and second mitotic divisions in the embryo. Our findings are broadly consistent with the recent proposal that meiosis-derived aneuploidy persists into embryogenesis, but that mitosis-derived aneuploidy triggers arrest (Wartosch et al., 2021). Furthermore, our results suggest that elongated spindles in zygotes and micronuclei in the two-cell embryo are visual markers of poor developmental outcomes, which could be useful for *in vitro* fertilization.

## MATERIALS AND METHODS

### Generation of mouse lines

All mice experimental protocols were approved by the Institutional Animal Care and Use Committee of the Stowers Institute for Medical Research (SIMR; Kansas City, MO, USA) and were performed accordingly. Mice were housed in a barrier facility with constant temperature, humidity and light at SIMR.

Mice were genotyped as described previously (de Vries et al., 2000; Lewandoski et al., 1997; Viny et al., 2015; Lan et al., 2004). *Smc3* heterozygous mice (*Smc3^fl/+^*) were maintained in the C57BL/6J genetic background (Viny et al., 2015). *Smc3^fl/fl^;Zp3-Cre* and *Smc3^fl/fl^;Gdf9-iCre* male mice were generated by crossing *Smc3^fl/+^* females with Tg(*Zp3-Cre*) (The Jackson Laboratory, #003651) or Tg(*Gdf9-iCre*) males (The Jackson Laboratory, #011062) in the C57BL/6J genetic background. Experimental mice were generated from heterozygous- or homozygous-floxed females with homozygous-floxed males positive for *Zp3-Cre* or *Gdf9-iCre*.

### Breeding trials

Fertility tests were carried out by crossing experimental females (6-8 weeks old) with wild-type C57BL/6J males. Female mice were checked for plugs every morning and each litter was recorded. Females were mated continuously for at least 6 months. The number of pups on the first day after parturition was counted as the litter size.

### l*acZ* reporter assay

Male *Gdf9-iCre* mice were crossed with female *Gt(ROSA)26Sor^tm1(lacZ)Cos^* mice (R26R, The Jackson Laboratory, #003474) and embryos were collected in PBS at 13.5 dpc. Gonads were dissected from each embryo and fixed at 4°C in fixative [1% formaldehyde, 0.2% glutaraldehyde, 2 mM MgCl_2_, 5 mM EGTA, in wash solution (0.02% NP40 in PBS)] for 10 min. Gonads were washed three times for 15 min each, in wash solution at room temperature. After washing, gonads were put in staining solution [5 mM K_3_Fe(CN)_6_, 5 mM K_4_Fe(CN)_6_.3H_2_O), 2 mM MgCl_2_, 0.01% sodium deoxycholate, 0.02% NP40 and 1 mg/ml X-gal] overnight and, the next morning, embryos were washed, imaged and stored in fixative at 4°C. Images were recorded using a Zeiss Axiovert microscope.

### Histological analysis of ovaries and follicle counting

Histological tissue sections of the whole ovaries were prepared as previously described (Singh et al., 2019). In brief, whole ovaries were fixed in Modified Davidsons fixative (Electron Microscopy Sciences; 64133-50) for 6 h at room temperature and then overnight at 4°C. Each ovary was dehydrated, embedded in paraffin blocks and then serially sectioned at a thickness of 5 μm. The slides were stained by H&E. Images were acquired by a VS120 Slide Scanner (Olympus) with a 40× objective. Primordial, primary, secondary and antral follicles were scored in every five sections throughout the entire ovary as previously described (Bristol-Gould et al., 2006; Duncan et al., 2017).

### *In vitro* maturation

To obtain fully grown GV-stage oocytes, 6- to 8-week-old females were injected with 5 IU pregnant mares' serum gonadotropin (PMSG; Genway Biotech; GWB-2AE30A). Prophase I-arrested GV-stage oocytes were isolated by physical dissection of ovaries in M2 medium (Sigma-Aldrich; MR-015-D) with 5 μM milrinone (Sigma-Aldrich; M4659). Isolated GV-stage oocytes were released from prophase I by briefly washing in Lebovitz's L-15 medium (Thermo Fisher Scientific; 11415-064). Oocytes were incubated in 90 μl drops of M16 medium (Sigma-Aldrich; M7292) covered with mineral oil at 37°C and 5% CO_2_. Metaphase II oocytes were obtained 14 h after incubation.

### *In vitro* culturing

To obtain zygotes, timed mating was performed by consecutive intraperitoneal injection of 5 IU PMSG followed by injection of 5 IU human chorionic gonadotropin (hCG; Sigma-Aldrich; C1063) 46 h later. Superovulated female mice were crossed with C57BL/6J male mice after hCG injection. The referenced time point was defined by hCG injection for all related experiments (hours post-injection; hpi). Females were sacrificed 20 hpi and oviducts were isolated by surgical dissection and placed in M2 medium. Zygotes were released from cumulus cells by a brief incubation with 500 µg/µl hyaluronidase/M2 medium (Sigma-Aldrich; H4272). Fertilized zygotes were selected based on visible pronuclei. Unfertilized oocytes were discarded, and only fertilized zygotes were incubated in ∼90 µl drops of KSOM medium (Sigma-Aldrich; MR-101-D) at 37°C with 5% CO_2_.

### Microinjection

To increase the yield of zygotes, superovulation was performed by injection of 7.5 IU CARD HyperOva (Cosmo Bio; KYD-010-EX) followed by injection of 7.5 IU hCG 46 h later. Zygotes were then isolated and incubated *in vitro*. The *Smc3* template was amplified from the Mouse SMC3 cDNA Clone in Cloning Vector (Genomics-online; ABIN4098669) by PCR using Q5 High-Fidelity DNA Polymerase (New England Biolabs; M0515), T3 and T7 primer pairs. mRNA was transcribed *in vitro* followed by 3′ tailing by ultra T7 mMessage mMachine kit (Invitrogen; AM1344) following the manufacturer's instructions. The purified mRNA was resuspended in either DEPC-treated water or TE buffer (10 mM Tris-HCl, 1 mM EDTA, pH 7.4) and then diluted to 10 ng/μl in TE buffer prior to microinjection of between 1 and 2 picoliters (pl).

### Fixation and immunofluorescence

To identify the cell cycle phase accurately, zygotes were pulsed with 100 mM EdU for 30 min. G1-, S- and G2-phase zygotes were classified by both the timing of fixation (G1, 20.5-22.5 hpi; S, 24-26 hpi; G2, 26-28 hpi) and the presence of EdU incorporation in the pronucleus. Only S-phase zygotes incorporated EdU into the pronucleus. Any zygote collected from the G1- or G2-phase time points with EdU signal was disqualified as a G1- or G2-phase pronucleus. To verify global RNA transcription, zygotes were pulsed with 1 mM 5-EU for 2 h before fixation.

To examine checkpoint activation, two-cell stage embryos were collected at 53.5 hpi (∼10% of wild-type embryos reached the four-cell stage at this time point). To introduce replication stress and exogenous DNA damage, zygotes were cultured with 4 mM hydroxyurea at 24 hpi for 4 h before fixation.

Metaphase zygotes were obtained from incubation with 10 µM ProTAME (Boston Biochem; I-440) to inhibit the anaphase-promoting complex (APC) for 3-4 h. Oocytes and zygotes were fixed with 2% paraformaldehyde (PFA)/PBS for 1 h at room temperature. Fixed samples were washed twice with blocking solution [0.3% bovine serum albumin (BSA), 1× PBS, 0.01% Tween-20 (Sigma-Aldrich; P1379), 0.02% sodium azide (NaN_3_)] for 5 min and then stored at 4°C. Samples were incubated in a permeabilization solution [0.3% BSA, 1× PBS, 0.1% Triton X-100 (Sigma-Aldrich; T9284), 0.02% NaN_3_] for 20 min and then washed twice with blocking solution for 5 min. Samples pulsed with EdU or 5-EU were processed following the guidelines of the Click-iT EdU Alexa Fluor 488 or 647 imaging kit (Invitrogen) as needed. Samples were immediately washed with blocking solution three times for 5 min each. anti-α-Tubulin 488 Alexa Fluor conjugate (Sigma-Aldrich; 16-232; 1:200), rabbit anti-γH2A.X (Cell Signaling; 9718; 1:500), mouse anti -γH2A.X (Sigma-Aldrich; 05-636; 1:500), RPA70 (Life Technologies; PA5-21976; 1:100), RAD51 (Sigma-Aldrich; PC130; 1:250), centromere serum (ImmunoVision; HCT-0100; 1:100), Chk1 (Cell Signaling; 2360; 1:100) and p-ATM (Active Motif; 39530; 1:100) primary antibodies were diluted in blocking solution and incubated with the samples overnight at 4°C. The next day, primary antibodies were washed out with blocking solution five times each for 10 min and incubated in Alexa Fluor 647 anti-human (Thermo Fisher Scientific; A-21445; 1:500), Alexa Fluor 647 anti-mouse (Life Technologies; A31571; 1:500), Alexa Fluor 594 anti-rabbit (Thermo Fisher Scientific; A-11072; 1:500), Alexa Fluor 594 anti-mouse (Thermo Fisher Scientific; A-11020; 1:500) or Alexa Fluor 488 anti-mouse secondary antibodies (Thermo Fisher Scientific; A-28175; 1:500) diluted in blocking solution for 2 h at room temperature. Secondary antibodies were washed out with blocking solution five times each for 10 min. Samples were mounted on glass slides in VECTASHIELD Antifade Mounting Medium with DAPI (Vector Laboratories; H-1200-10). Image acquisition was performed on a Carl Zeiss LSM-710 confocal microscope with a 63× oil immersion objective and *z*-stacks were taken every 0.5 µm.

### Chromosome spreads

Slides of chromosome spreads were prepared as described before (Hodges and Hunt, 2002). In brief, zona pellucida was removed in acidic M2 medium. Oocytes in metaphase II or zygotes in metaphase were briefly washed with M2 medium and then deposited on a glass slide with ∼70 µL drops of spread solution [1% PFA, 0.15% Triton X-100, 3 mM dithiothreitol in distilled H_2_O (pH 9.5)]. Slides were incubated in a humid chamber for 1 h and then dried overnight. Slides could be stored at −20°C before staining. Debris was washed from slides using 0.4% Kodak Photo-Flo 200 solution twice for 5 min, 1× PBS for 5 min and 1× PBS-0.1% Tween-20. Slides were incubated in the blocking solution for 1 h. Subsequent immunofluorescence was performed as mentioned earlier, using an Alexa Fluor 488 anti-human secondary antibody (Thermo Fisher Scientific; A-11013; 1:500).

### Live cell imaging microscopy

Live zygotes were incubated with 100 nM SIR-DNA dye (Cytoskeleton; CY-SC007) diluted in ∼90 µl KSOM medium for 30 min. Stained embryos were then transferred to an Interchangeable Coverglass Dish (Bioptechs; 190310-35) with a 30 mm diameter, 1.5-thickness coverslip (Bioptechs; 30-1313-0319) in SIR-DNA/KSOM medium. Image acquisition was performed on a Nikon 3PO spinning disc microscope with a 40× water objective. Microscopy was controlled by Nikon's Confocal NIS-Elements Package. Time-lapse images were acquired with ∼13 *z*-sections of 3 µm every 3 min.

### Western blot analysis

Fully grown GV-stage oocytes from each group were isolated as mentioned earlier, and briefly washed with L-15 medium. Then, 30 oocytes per tube were collected in 10 µl of L-15 medium and snap-frozen in liquid nitrogen. Frozen oocytes were stored at −80°C before testing. Proteins were extracted using Bio-Rad sample buffer (161-0747) with 2-mercaptoethanol (Sigma-Aldrich; M7522) at 95°C for 10 min and then centrifuged at 16,000 ***g*** for 2 min; the supernatant was then collected. Proteins were separated using Bio-Rad 4-15% gradient gels (456-1084) and blotted on a PVDF membrane (GE Healthcare; 10061-494). Blots were blocked with 0.3% ECL Prime Blocking Reagent (GE Healthcare; RPN418) in Tris-buffered saline supplemented with 0.01% Tween (TBSTw) for 1 h. SMC3 (Abcam; ab9263; 1:2000) and γ-tubulin (Abcam; ab11316; 1:1000) primary antibodies were diluted in 0.3% blocking buffer at 4°C overnight. Blots were washed with TBSTw three times for 10 min each and then incubated with anti-rabbit or anti-mouse HRP-conjugated secondary antibodies (GE Healthcare; NA931V and NA934V, respectively; 1:5000). Blots were washed with TBSTw three times each for 10 min and then developed with a ECL Prime Western Blotting Detection Reagent kit (GE Healthcare; RPN2232).

### Quantification, foci counting and imaging analysis

Image analysis was performed using open-source Fiji software (Schindelin et al., 2012). The intensities of the western blots were quantified using the gel analysis method outlined in ImageJ. Foci quantification was performed as described previously with minor modifications (Cai et al., 2009). Briefly, image stacks were background subtracted by a rolling radius of 1.32 µm or 3.86 µm, and foci counting was performed by analysis of particles >0.5 µm^2^ or >0.01 µm^2^ corresponding to the cell size covered by the entire *z*-stack range. Thresholds were kept at (20, 255) and (150, 255) within all experiments.

To quantify the L/W ratio of the metaphase plate and spindle, the *z*-projections of stack images were Gaussian blurred by a radius of 1.37 µm in the live cell imaging and by 1.5 µm in the immunostaining of metaphase zygotes. Thresholds were automated within all experiments to visualize the boundaries for length measurement.

The quantification of interkinetochore distance was performed as previously described (Cahoon et al., 2017). In brief, only image stacks with an appropriate orientation were selected for the quantification. All distinguishable centromere pairs were counted in each *xy* image. The line profile of CREST signal along the *x*-axis was generated from aligning the center of two peaks. The average profiles were fitted into a two-Gaussian model to determine the width of the two peaks.

### Statistics

The statistical parameters and tests used are described in the figure legends. Statistical analysis was performed using Microsoft Excel 365 and R studio. The parametric two-tailed, unpaired Student's *t*-test and one-way ANOVA test with the Tukey HSD test were performed for datasets, whereas the nonparametric unpaired Mann–Whitney *U*-test was used for datasets not passing the Iglewicz and Hoaglin's robust test for multiple outliers within a *z* score of 3.5. Fisher's exact test was performed to examine the contingency table datasets whereas the Chi-Square test was used to examine the difference between categorical variables.

## Supplementary Material

Supplementary information

Reviewer comments
