# Supplementary material for: Maternal Smc3 protects the integrity of the zygotic genome through DNA replication and mitosis
Source: Development. 2021 Dec 22;148(24):dev199800. doi: 10.1242/dev.199800 (PMC8722392; doi:10.1242/dev.199800)
Supplement: Supplementary information [file develop-148-199800-s1.pdf]

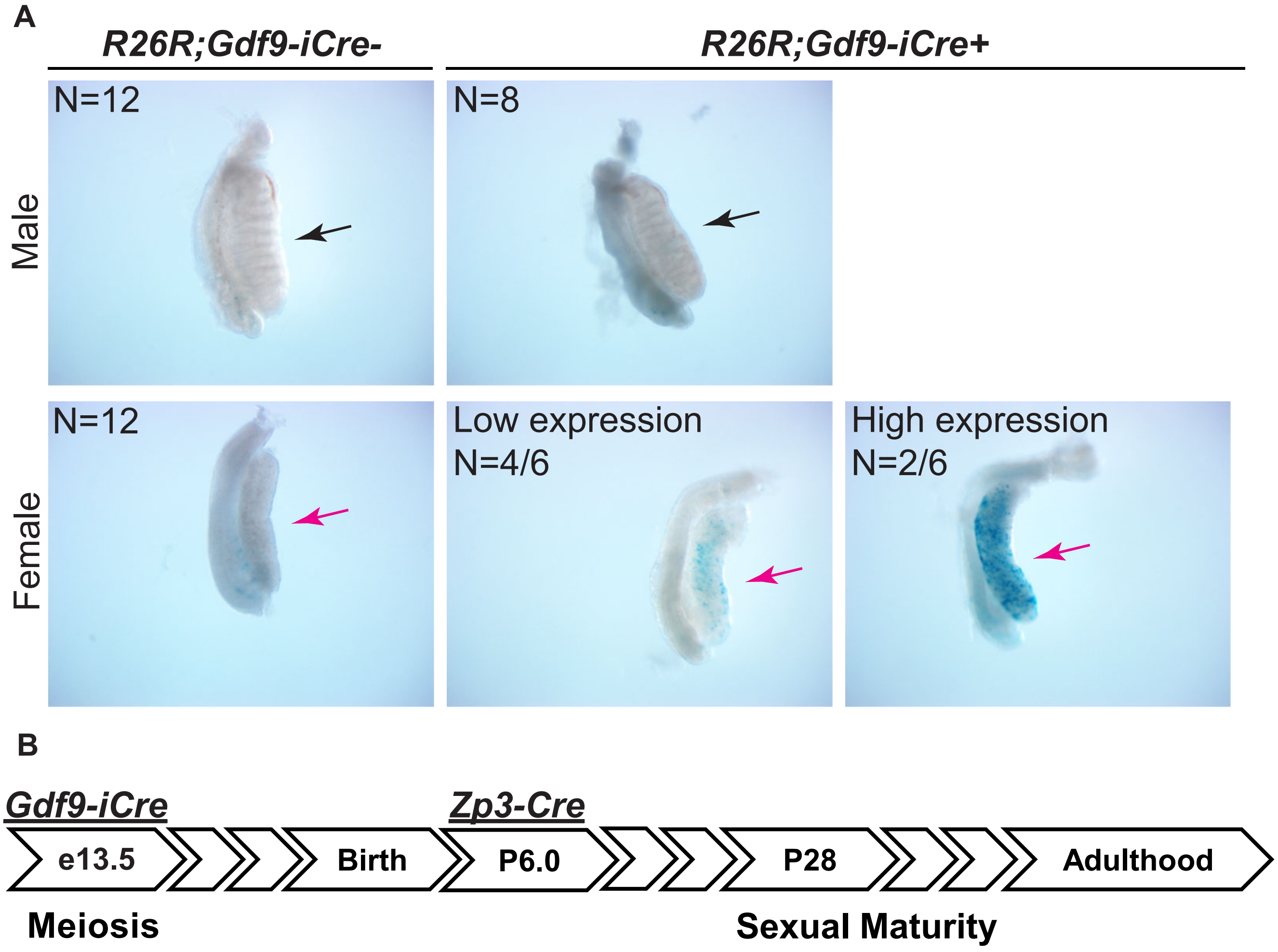

Fig. S1. *Gdf9-iCre* is expressed in the female gonad at 13.5 *dpc*. A) *Gt(ROSA)26Sor$^{tm1(LacZ)Cos}$* (known as *R26R*) females were crossed with *Gdf9-iCre$^+$* males The STOP sequence is removed and the downstream lacZ gene is expressed in cells/ tissues where cre is expressed. β-gal staining in mouse gonads at 13.5 *dpc* was detected using X-gal. Male gonads and *cre* negative gonads were tested as negative controls; female gonads with *Cre$^+$* showed β-gal staining. *n*=12 for *R26R* male gonads, 8 for *R26R Gdf9-iCre$^+$* male gonads, 12 for *R26R* female gonads, and 6 for *R26R Gdf9-iCre$^+$* female gonads. Black arrows indicate representative male gonads. Magenta arrows indicate representative female gonads. Sex is determined by morphology of the gonad and genotyping of the embryo. **B)** A schematic of Cre driver expression in relation to mouse development.

Development • Supplementary information

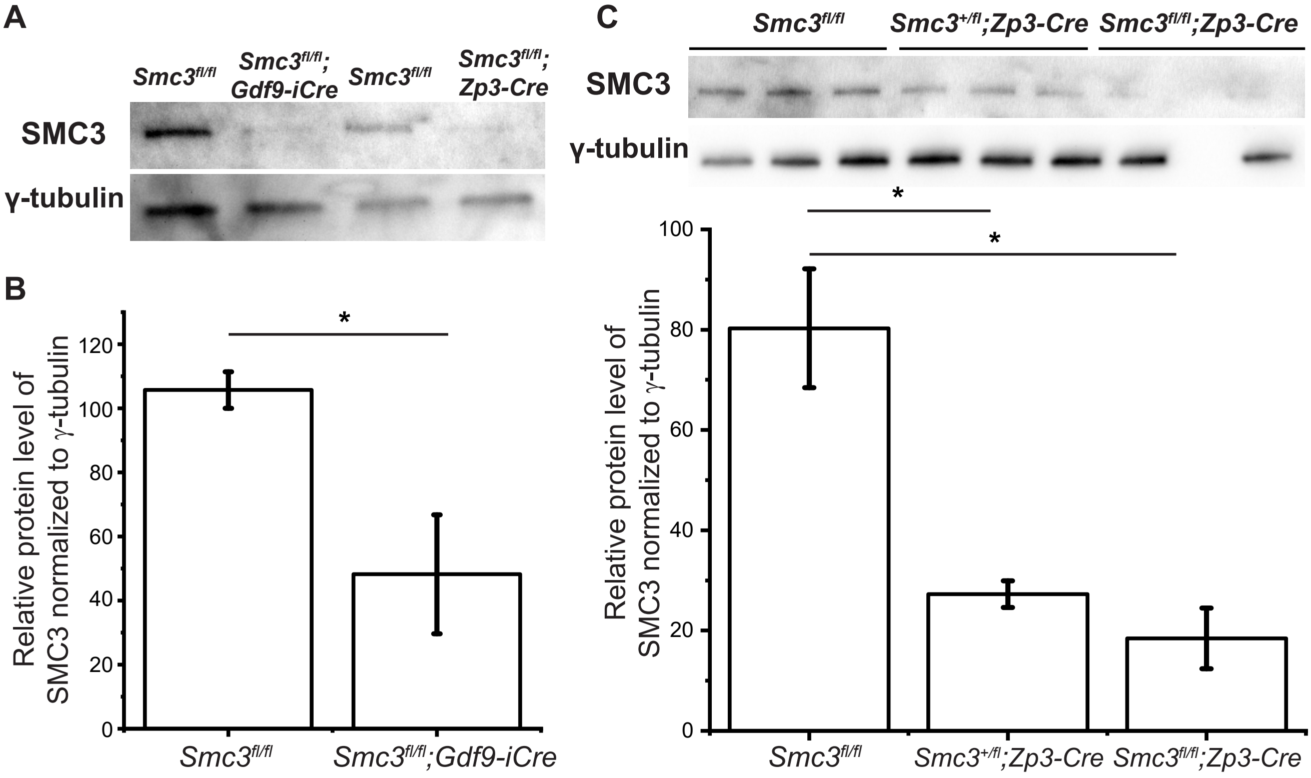

Fig. S2. Maternal SMC3 deprived from oocytes by *Gdf9-iCre and Zp3-Cre* drivers. Related to Fig. 1. Fully grown germinal vesicles (GV) stage oocytes were collected and snap frozen from ovaries of PMSG-stimulated adult female mice (6-8-week-old). The level of SMC3 was examined and γ-Tubulin was used as the loading control. *n*=30 oocytes per lane. **A)** A representative western blot is shown for oocytes from *Smc3$^{fl/fl}$*, *Smc3$^{fl/fl}$;Gdf9-iCre*, and *Smc3$^{fl/fl}$;Zp3-Cre* female mice. **B)** Quantification of the level of SMC3 in *Smc3$^{fl/fl}$* and *Smc3$^{fl/fl}$;Gdf9-iCre* oocytes is shown (*n, Smc3$^{fl/fl}$*=4, *Smc3$^{fl/fl}$;Gdf9-iCre*=5). **C)** Western blots and their corresponding quantification from oocytes derived from *Smc3$^{fl/fl}$* (wild type), *Smc3$^{+/fl}$;Zp3-Cre* (heterozygous), and *Smc3$^{fl/fl}$;Zp3-Cre* (homozygous) female mice is shown (*n*, *Smc3$^{fl/fl}$*=4, *Smc3$^{+/fl}$;Zp3-Cre*=3, *Smc3$^{fl/fl}$;Zp3-Cre*=3). The chemiluminescence values quantified by ImageJ are an imperfect reflection of what is observed by eye. Statistical data are represented with mean ± standard deviation. An unpaired two-tailed student t-test was used to determine statistically significant differences between genotypes. *, $p < 0.05$.

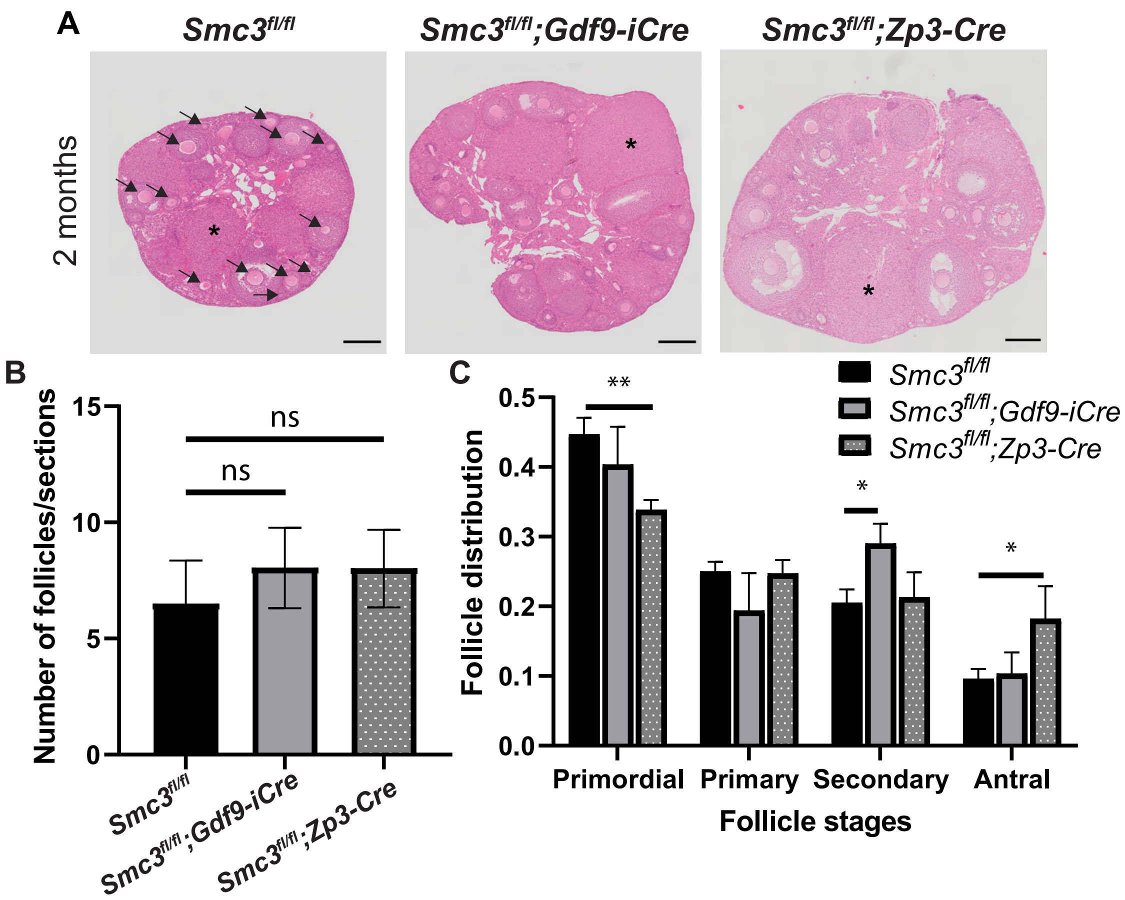

Fig. S3. Removal of maternal *Smc3* from oocytes by either the *Gdf9-iCre* or *Zp3-Cre* drivers does not reduce ovarian reserve. Follicles were scored and categorized in histological sections from the entire ovary. *n*=3 mice for each genotype. **A**) Hematoxylin and eosin staining of histologic sections was performed on ovaries from 2-month-old mice of each genotype. Arrows indicate follicles consisting of an individual oocyte surrounded by granulosa cells. Asterisks indicate the corpus lutea. **B)** Quantification of the total number of follicles in ovaries from 2-month-old *Smc3$^{fl/fl}$*, *Smc3$^{fl/fl}$;Gdf9-iCre*, and *Smc3$^{fl/fl}$;Zp3-Cre* female mice shows no difference in number as a function of genotype. **C)** The distribution of follicle stages in ovaries from 2-month-old mice is shown as a function of genotype. An unpaired two-tailed student t-test was used to determine statistically significant differences between genotypes. *, p<0.05. **, p<0.01. Scale bar, 100 μm.

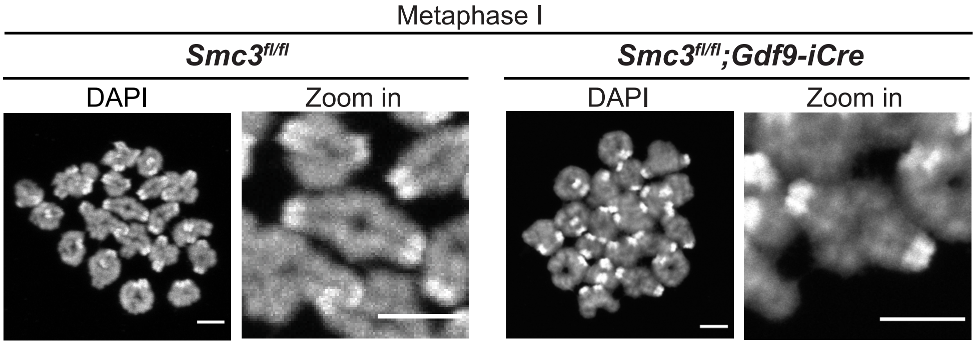

Fig. S4. Meiotic sister chromatid cohesion was not affected in *Smc3*$^{\Delta/\Delta}$*Gdf9-iCre* oocytes in metaphase I. Related to Fig. 1. Chromosome spreads were prepared as described in **Fig. 1**. Intact bivalents showed that sister chromatid cohesion was comparable between *Smc3*$^{fl/fl}$ and *Smc3*$^{fl/fl}$*;Gdf9-iCre* oocytes (*n, Smc3*$^{fl/fl}$=2, *Smc3*$^{fl/fl}$*;Gdf9-iCre*=6). Images were captured by confocal microscope. Scale bar, 5 µm.

Development • Supplementary information

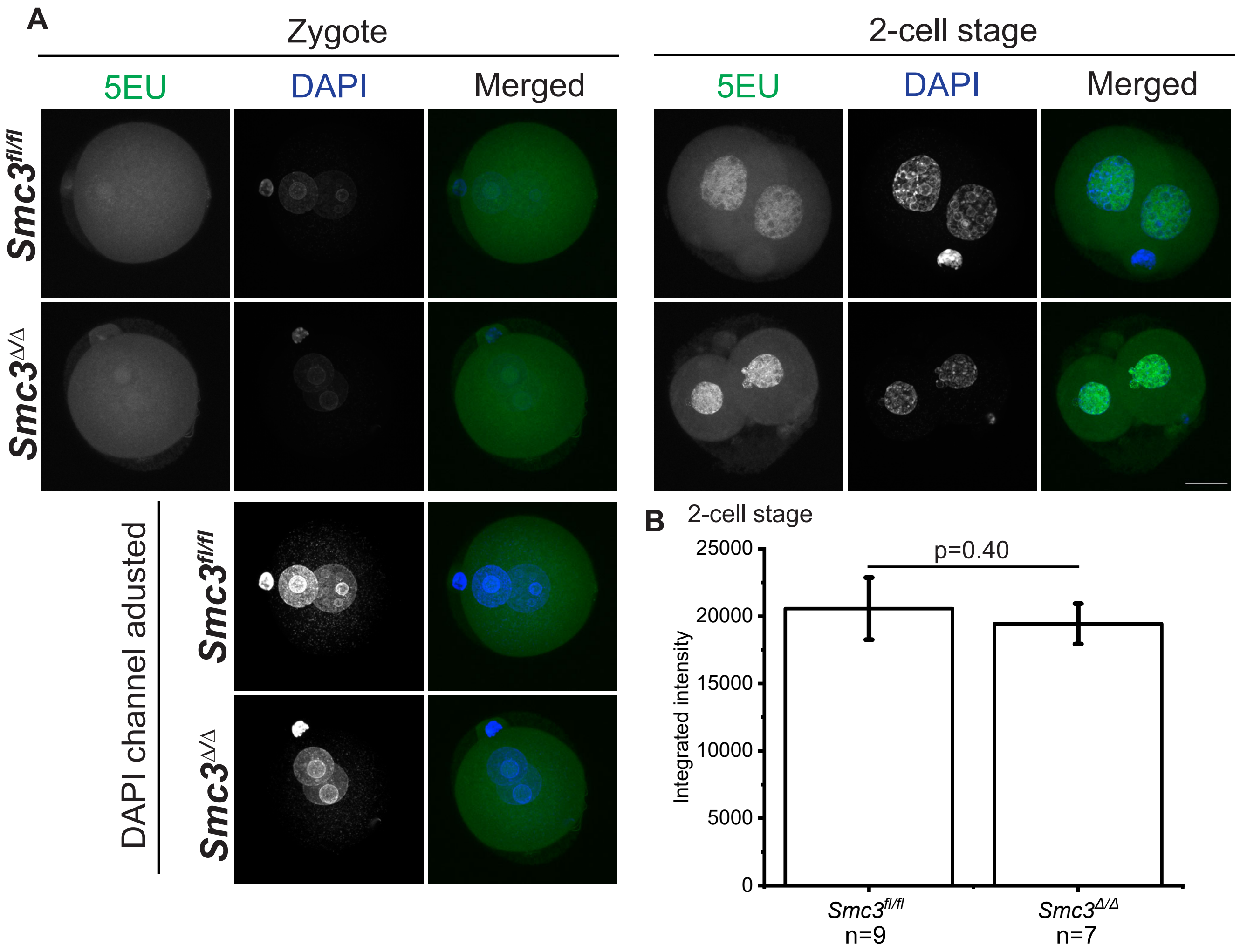

Fig. S5. Zygotic genome activation occurred with normal timing when *Smc3* is depleted. Zygotes from adult *Smc3*$^{fl/fl}$ and *Smc3*$^{\Delta/\Delta}$ *cKO* female mice were incubated with 5EU 2 hours before fixation. **A)** Samples were developed by Click-iT assay (green) and nuclei were labeled with DAPI (blue) *(n, Smc3*$^{fx/fx}$ zygote=10, *Smc3*$^{\Delta/\Delta}$ zygote=12, *Smc3*$^{fx/fx}$ 2-cell stage=9, and *Smc3*$^{\Delta/\Delta}$ 2-cell stage=7). **B)** Integrated intensity of 5EU signal from nuclei was quantified as an indicator of global RNA transcription. Statistical data are represented with mean ± standard deviation. A student t-test was used to determine statistically significant differences between genotypes. Scale bar, 20 µm.

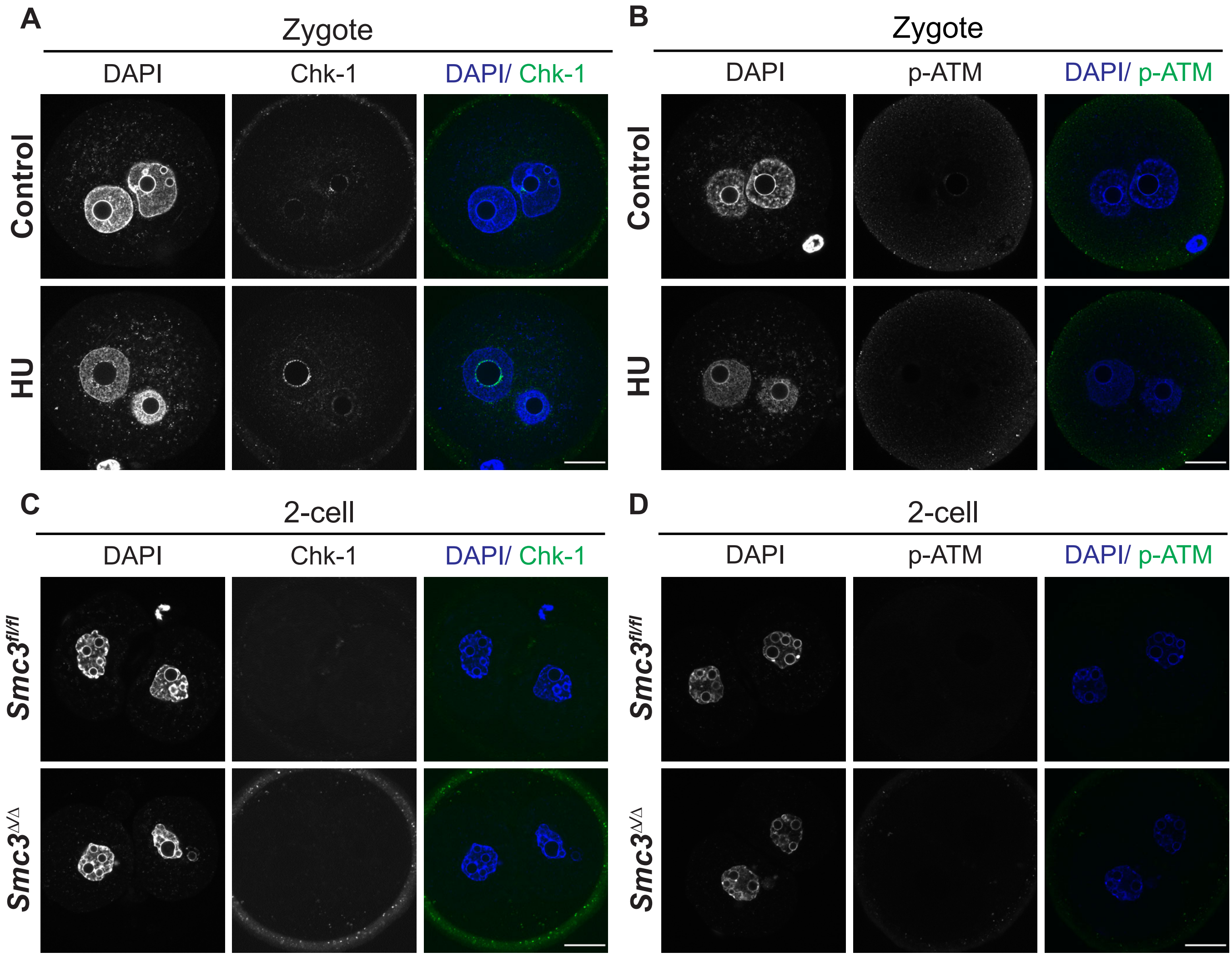

Fig. S6. Canonical ATR and ATM checkpoints are not activated in mutant embryos. A-B) Zygotes from wildtype females were cultured with hydroxyurea (HU) to introduce replication stress. Total Chk1 **(A)** and p-ATM **(B)** were labeled in 28 hpi zygotes (*n*, *Smc3*$^{fx/fx}$=6, *Smc3*$^{\Delta/\Delta}$=6 for each immunostaining). **C-D)** Immunostaining of Chk1 **(C)** and p-ATM **(D)** in *Smc3*$^{fx/fx}$ and *Smc3*$^{\Delta/\Delta}$ embryos at 2-cell stage (*n*, *Smc3*$^{fx/fx}$=11, *Smc3*$^{\Delta/\Delta}$=24 for each immunostaining). Scale bar: 20 μm.

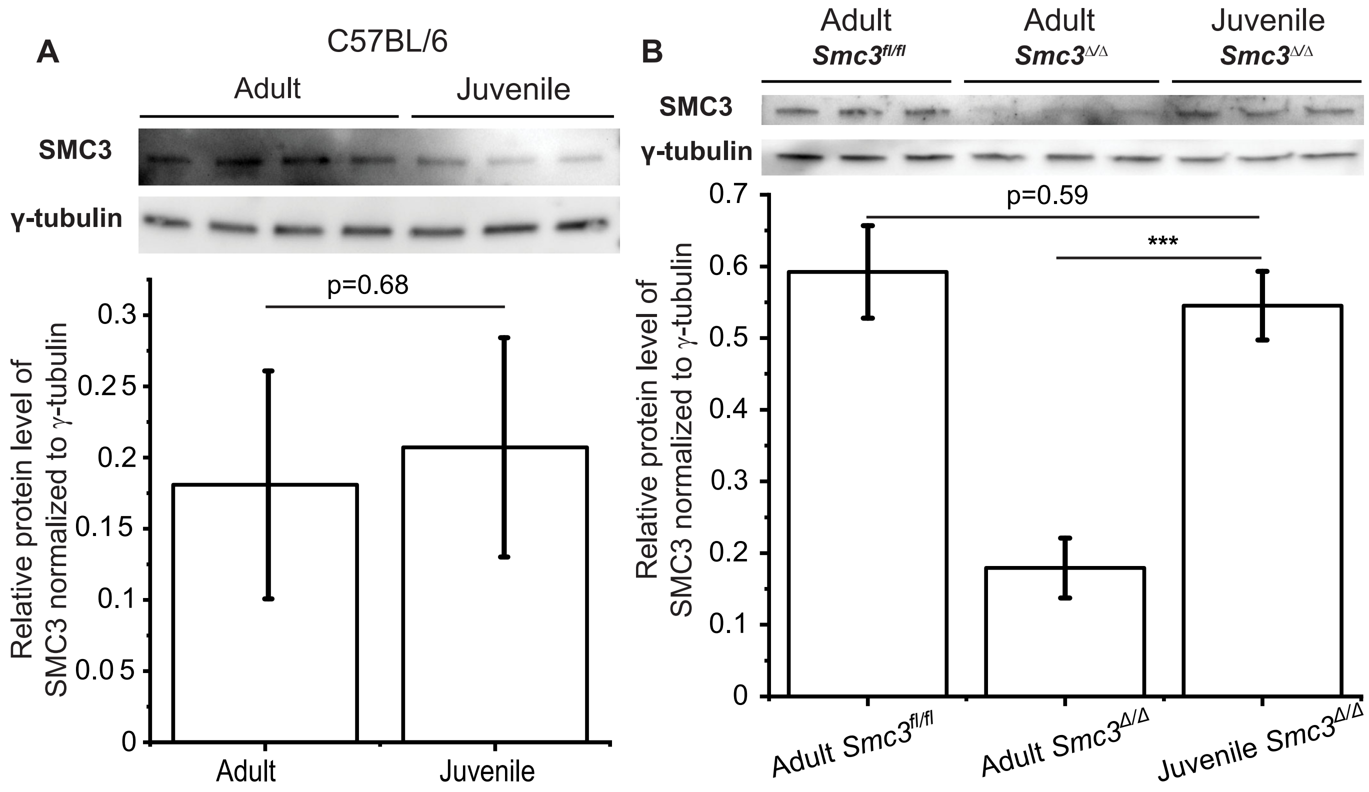

Fig. S7. SMC3 protein levels are sustained in mutant oocytes from juvenile females. Related to Fig. 6. Fully grown GV stage oocytes from adult or juvenile females of the indicated genotypes were collected as described in **Fig. S2**. The level of SMC3 was examined and γ-Tubulin was used as the loading control. *n*=30 oocytes per lane. **A)** Analysis of the western blot of oocytes from adult and juvenile C57BL/6 female mice (*n*=4 replicates for adult and 3 for juvenile). **B)** Analysis of the western blot of oocytes from adult *Smc3*$^{fl/fl}$, adult *Smc3*$^{fl/fl}$*;Zp3-Cre*, and juvenile *Smc3*$^{fl/fl}$*;Zp3-Cre* female mice is shown (*n*=3 per genotype). Statistical data are represented with mean ± standard deviation. An unpaired two-tailed student t-test was used to determine statistically significant differences between genotypes. *, $p < 0.05$. ***, $p < 0.001$.

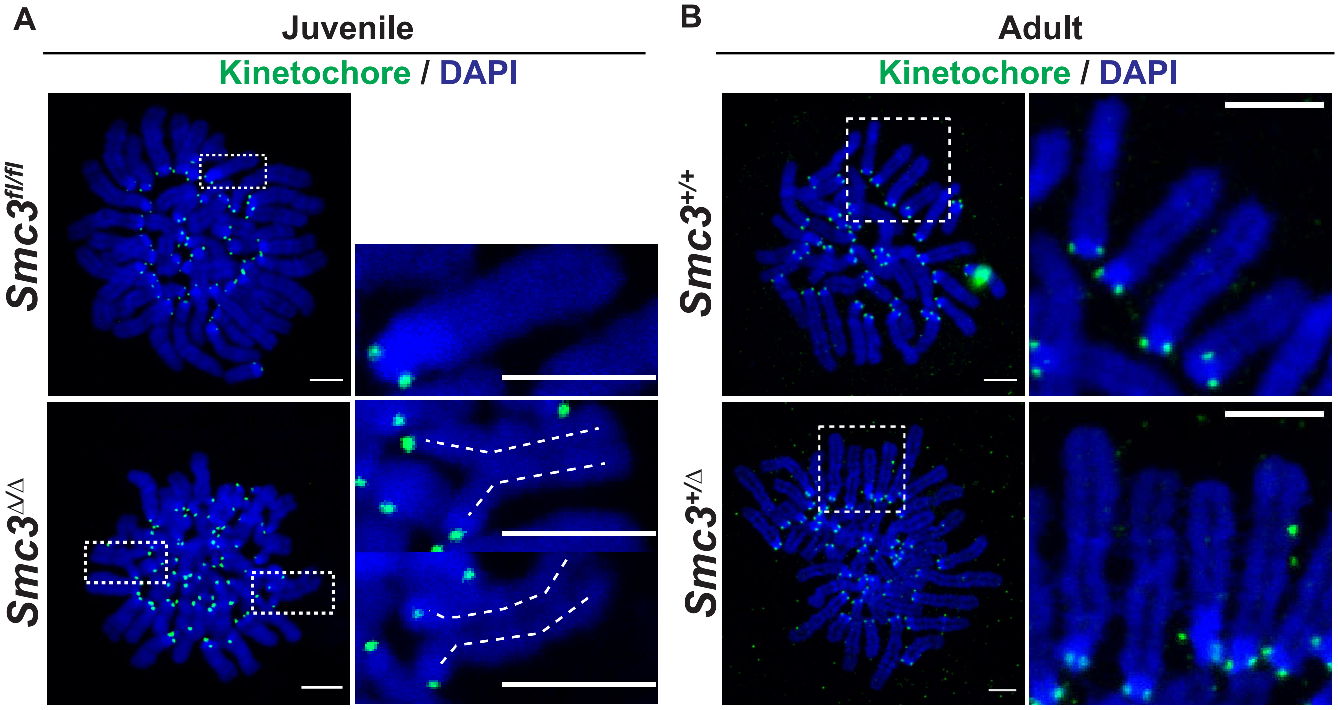

Fig. S8. Loss of maternal *Smc3* disrupts the chromosome structure in zygotes from juveniles. Related to Fig. 7. Zygotes in metaphase were prepared from $Smc3^{+/+}$, $Smc3^{fl/fl}$, $Smc3^{+/fl}$;*Zp3-Cre* ($Smc3^{+/\Delta}$) and $Smc3^{\Delta/\Delta}$ *cKO* female mice as described in **Fig. 7**. Chromosome spreads were prepared and labeled with CREST (kinetochores, green) and DAPI (DNA, blue) (*n,* juvenile $Smc3^{fl/fl}$=1, juvenile $Smc3^{\Delta/\Delta}$=4, adult $Smc3^{+/+}$=4, and adult $Smc3^{+/fl}$;*Zp3-Cre* ($Smc3^{+/\Delta}$)=7). Dotted squares indicate the selected area which is shown at increased magnification in the right panel. Dotted lines trace the chromosome arms. Scale bar, 5 μm.

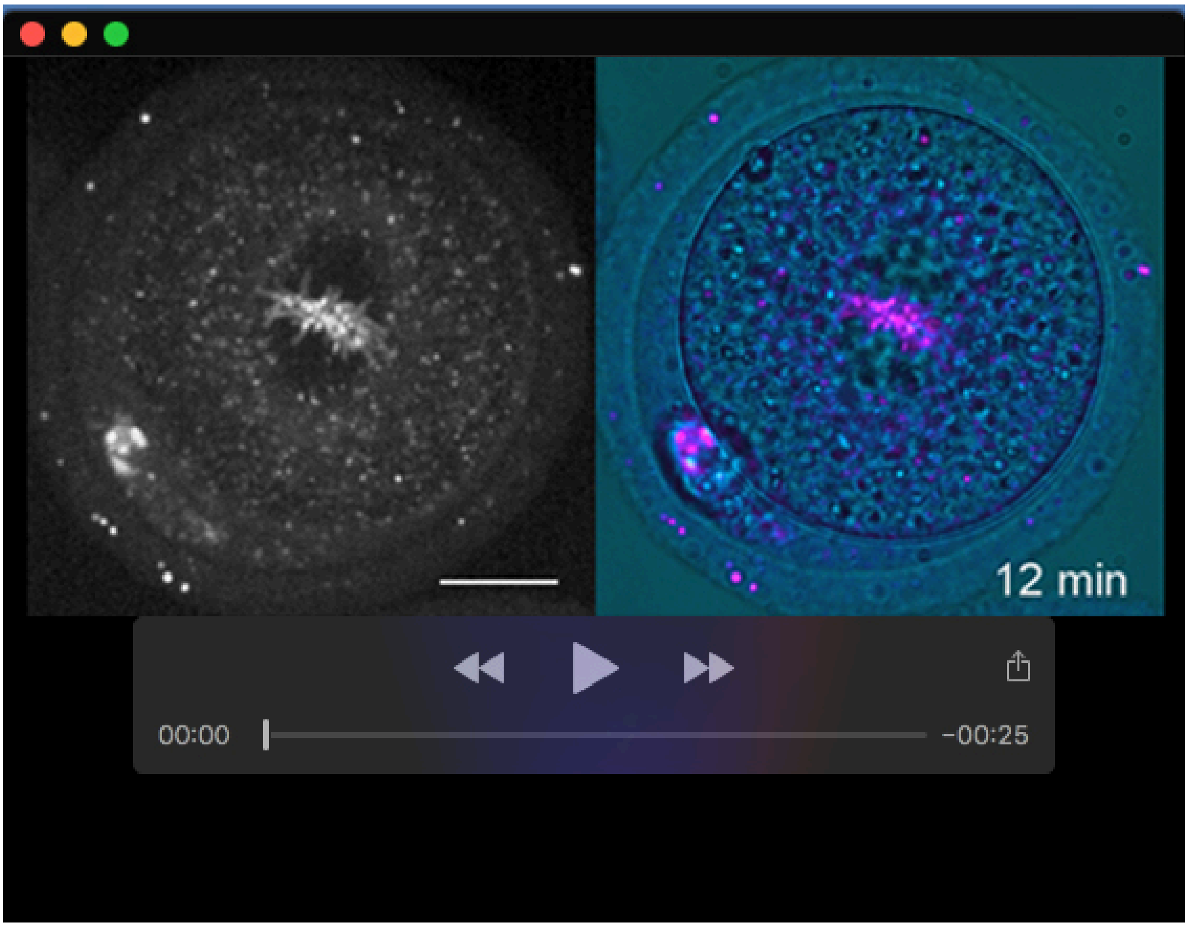

Movie 1. Video of live cell imaging of SIR-DNA dye recording the first mitotic division in *Smc3*$^{fl/fl}$ zygotes. Related to Fig. 4C-E. Left panel shows the SIR-DNA channel and the right panel shows the merged stack of the SIR-DNA channel (magenta) and the widefield channel (cyan). Scale bar, 10 µm.

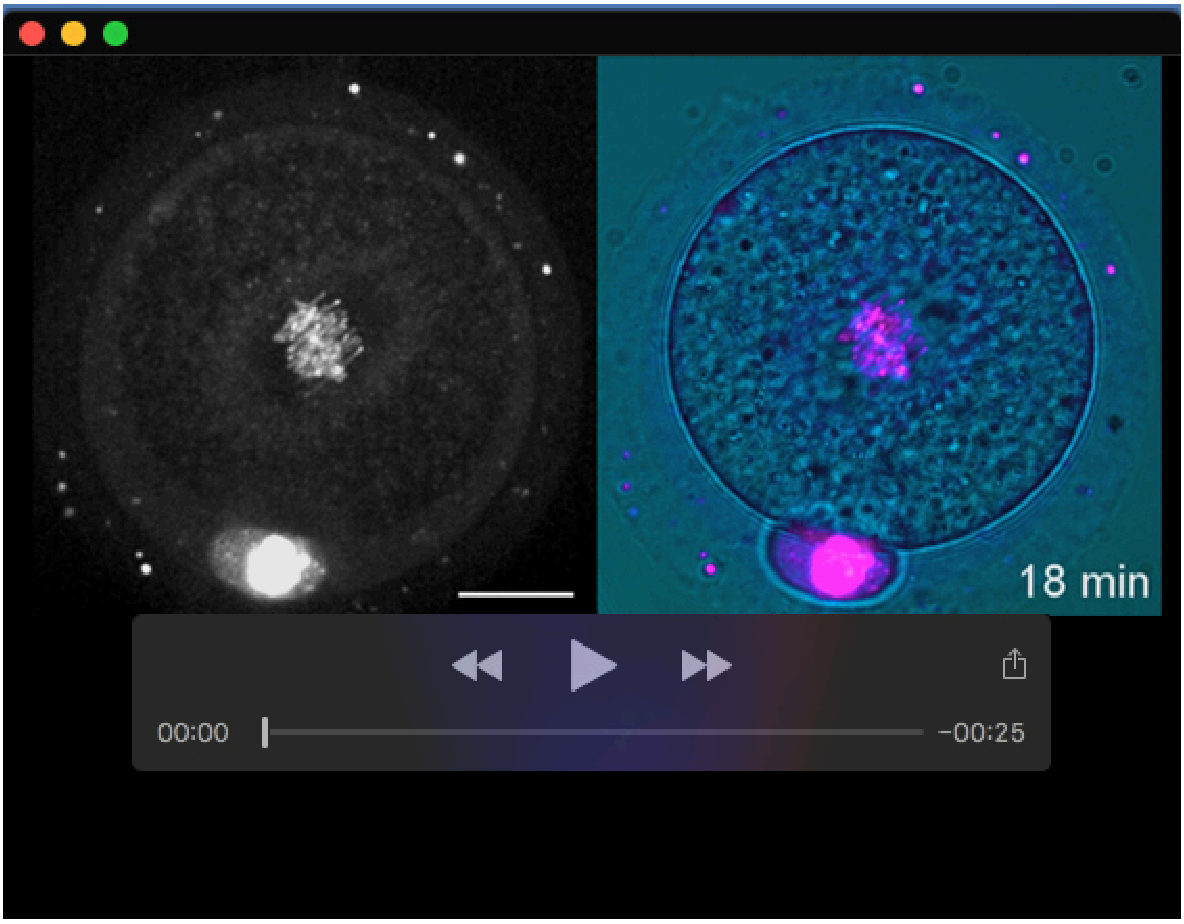

Movie 2. Video of live cell imaging of SIR-DNA dye recording the first mitotic division in *Smc3*$^{\Delta/\Delta}$ *cKO* zygotes. Related to Fig. 4C-E. Left panel shows the SIR-DNA channel and the right panel shows the merged stack of the SIR-DNA channel (magenta) and the widefield channel (cyan). The magenta arrow indicates the micronucleus formed from lagging chromosomes. Scale bar, 10 µm.
