# Supplementary material for: Maternal Smc3 protects the integrity of the zygotic genome through DNA replication and mitosis
Source: Development. 2021 Dec 22;148(24):dev199800. doi: 10.1242/dev.199800 (PMC8722392; doi:10.1242/dev.199800)
Supplement: Reviewer comments [file develop-148-199800_review_history.pdf]

Maternal *Smc3* protects the integrity of the zygotic genome through DNA replication and mitosis

Wei-Ting Yueh, Vijay Pratap Singh and Jennifer Gerton
DOI: 10.1242/dev.199800

Editor: Swathi Arur

Review timeline

Original submission:	12 May 2021
Editorial decision:	17 June 2021
First revision received:	21 October 2021
Editorial decision:	8 November 2021
Second revision received:	18 November 2021
Accepted:	22 November 2021

Original submission

First decision letter

MS ID#: DEVELOP/2021/199800

MS TITLE: Maternal Smc3 protects the integrity of the zygotic genome through DNA replication and mitosis

AUTHORS: Wei-Ting Yueh, Vijay Pratap Singh, and Jennifer Gerton

I have now received all the referees' reports on the above manuscript, and have reached a decision. All three reviewers agree that the study provides novel insights into the role of SMC3 during embryonic development and potentially as a maternal effect gene. However, the referees have some significant concerns that will need to be addressed before we can consider publication. Specifically, as reviewer 1 suggests, the role of Smc3 upon deletion with Gdf9-Cre requires more substantiation. In line with this point, an analysis of the Smc3 with immunofluorescence to assess the localization to chromosomes would be more powerful than a bulk Western Blot which assesses total protein load. Reviewer 1 also suggests analysis of oocyte numbers directly with histology to definitively assess the impact of Smc3 deletion on ovarian reserve. Reviewer 3, similarly suggests a direct assessment of the claim that Smc3 functions as a maternal effect gene by experimentally rescuing the developmental arrest phenotype. Reviewer 2 provides suggestions to improve the manuscript textually and to increase the scholarliness of the work. If you are able to revise along the lines suggested by the reviewers, I would be happy to receive a revision.

Please attend to all of the reviewers' comments and ensure that you clearly highlight all changes made in the revised manuscript. Please avoid using 'Tracked changes' in Word files as these are lost in PDF conversion. I should be grateful if you would also provide a point-by-point response detailing how you have dealt with the points raised by the reviewers in the 'Response to Reviewers' box. If you do not agree with any of their criticisms or suggestions please explain clearly why this is so.

© 2021. Published by The Company of Biologists under the terms of the Creative Commons Attribution License (https://creativecommons.org/licenses/by/4.0/).

Reviewer 1

Advance summary and potential significance to field

This is an interesting manuscript from Yueh et al. that evaluates the requirement for the cohesin subunit Smc3 during meiosis and early embryogenesis. They take a mouse oocyte knockout approach to tackle this biological problem. Unlike deletion of other cohesin subunits, they find that meiosis in oocytes lacking Smc3 is normal, but there are significant abnormalities in the early embryo leading to a 2 cell embryo arrest and sterility. They very nicely detail accumulation of DNA damage after the 1st S phase in the embryo, accumulation of micronuclei, lagging chromosomes and loss of cohesion. Finally, they document that some of these defects depend on maternal age, as juvenile females do not have a 2 cell arrest, but do have some damage accumulation and appearance of abnormal chromosome configuration indicating loss of cohesion occurring.

Comments for the author

There are many aspects of this story that I find strong and are exciting findings (ie- the ones I just described). However, there are parts that are not strong and detract from the story that I detail below.

1. Use of Gdf9-Cre: The authors begin the MS by comparing KO of Smc3 using 2 Cre drivers and attempt to demonstrate that the Gdf9 Cre is excising Smc3 during meiotic S phase. The data presented is confusing and not convincing to this reviewer. This is because the authors show 2 BGal stained gonads with varying expression levels (1 high, 1 low), suggesting that the expression of Gdf9 at this time must vary and is likely mosaic. How many gonads were weak vs strong expression in their sampling? Do all the oocytes in the gonads express the Cre? They should document that SMC3 is being removed at this time. They also mention 2 time points of S phase; e13.5 and e11.5. Which is it? I thought it was the earlier time point? If the timing of S phase is not clear, and if it is not clear that all oocytes are excising Smc3 at this time, then it is not convincing that the Gdf9 model is really that different in biological timing than the Zp3. Then, this model is not used for the rest of the MS because the phenotype is the same as the Zp3 model. So, I find use of this model and the time spent building this part of the MS distracting. I strongly recommend removing it; the paper will be improved without it. If the authors elect to keep it, they will need to do several more experiments to make it convincing for something that overall does not contribute to the main point of the MS.

2. Calling this strain a KO: The level of KO via western blotting is concerning. The quantification shows that there is still about 20% of the protein remaining in GV oocytes, which makes it a depletion, not a knockout. If they were to collect MII eggs, would the deletion be better (due to turnover during MI) and therefore explain why the embryo is impacted and not the oocyte? The reason why it concerns me is because the authors make the claim that it is not required for meiosis. But, formally, this cannot be stated because it is not a true KO. It is possible that the remaining 20% supports MI but not the embryo or it is possible that by Met II there is much less than 20% explaining why the embryos are impacted but not the oocyte. Therefore, the claim that this is a maternal effect gene is over-interpreted. Another possibility is that there is no Smc3 loaded, and the 20% is cytoplasmic and not functional. Can the authors try performing IF for SMC3 during MI? The functional population is much more important and would be more convincing as a maternal effect gene if the remaining is simply not at the right place (ie- not functional). Alternatively, the authors could try siRNA or Trim-Away depletion and support the claim of not being required during MI.

3. Age: Can the authors explain why 2 weeks makes a difference in the 2 cell arrest phenotype? Does this reflect a difference on hormonal impact of oocytes that are not ovulated in the 1st wave?

4. Reserve: The table 2 indicates fewer zygotes isolated from KOs. Do they ovulate fewer eggs or have fewer GV oocytes? If so, maybe the oocytes impacted by reduction of Smc3 are removed from the ovarian reserve during growth? These basic parameters should be evaluated because the authors could be missing an important part of the phenotype. This is because the authors claim only an effect on the embryos, but perhaps they are missing the effect on oocytes because they die.

© 2021. Published by The Company of Biologists under the terms of the Creative Commons Attribution License (https://creativecommons.org/licenses/by/4.0/).

5. Western: The authors should show the full length images of westerns.

6. A few comments in the text I recommend changing:
Line 64: “How genome integrity at this stage is monitored by checkpoints is an open question” I would modify because the authors don’t address any checkpoints.
Line 90- also brings in checkpoints, but really this isn’t a checkpoint paper so I find it confusing
Line 122- also brings up a checkpoint- same confusion.
Line 95- mentions “the same Zp3-cre driver” but this is the first time that is mentioned. Needs to be edited.
Line 130- How is the Cre improved? This is the standard cre to my knowledge.
Line 151- describes the het levels being intermediate. The graph shows Smc3 levels being closer to KO so I find this point not accurate.
Figure 1B- are these MI or MII spindles?
Line 301- I think you mean parental, not paternal.

Reviewer 2

Advance summary and potential significance to field

The study provides novel insight into the working mechanism of maternal Smc3 in early embryonic development. Using a plethora of cutting-edge experimental techniques, the research demonstrates that oocyte-stored SMC3 is required to support the integrity of the zygotic genome during the very first round of DNA replication and sister chromatid segregation to successfully pass through the first and second mitotic divisions in the embryo, making cohesin a key protector of the zygotic genome.

Comments for the author

1. Please provide full forms of all abbreviations when first used in manuscript body and abstract. This would also help non-expert readers.
2. Please specify the number of replicates conducted for each method, and if results were consistent across all replicates. This is important to ensure reproducibility.
3. Please double-check any minor typos/errors in spelling, punctuation, grammar etc. (for e.g., Western and not 'western' blot, use 'z' or 'Z' confocal image-
stacks consistently, etc.).
4. In the abstract and at the end of conclusions, it would be great to include the overall, big-picture application and significance of the work.
5. Please discuss any limitations of the study and recommend potential future directions to overcome them.
6. Methods - Statistics - please include Fishers exact test suitably.
7. Figures 3B and 6C - is it possible to specify the units for the intensities?
8. Please double-check and clarify all relevant controls for each of the methods.
9. To increase the cross-domain visibility and citability of this paper, additional works on some relevant conserved proteins like the 14-3-3 (YWHA), which are known to be abundant in mammalian oocytes and and eggs (including meiotic spindle) and interact isoform-specifically with multiple other factors to regulate molecular cross-talks in oocyte maternal effects, developmental competence, oocyte maturation, and/or early embryogenesis, could be suitably implicated by citing the following:
a) https://link.springer.com/article/10.1186/1756-0500-5-57
b) https://doi.org/10.1186/1471-213X-13-10 c) https://link.springer.com/article/10.1186/s12861-019-0200-1
d) https://www.tandfonline.com/doi/pdf/10.4161/cc.24991

Reviewer 3

Advance summary and potential significance to field

In this manuscript, Yueh et al. found that maternal SMC3 protein is required for maintain of the zygotic integrity and its chromatin segregation, and that depletion of maternal Smc3 gene results in

© 2021. Published by The Company of Biologists under the terms of the Creative Commons Attribution License (https://creativecommons.org/licenses/by/4.0/).

developmental arrest at the 2-cell stage (although such phenotype is dependent on maternal age). Overall, the characterization of SMC3 as a maternal effect gene is significant advance to the field of mammalian embryonic development. Moreover, most data is of good quality and the experiments are clearly and logically presented. However, there are several aspects of the manuscript that need to be improved/strengthened as follows:

Comments for the author

Major points:
1) My main concern is about the developmental ability of zygotes collected from juvenile (3-4 weeks old) Smc3Δ/Δ female. The authors argue that there is more SMC3 protein remaining in the juvenile Smc3Δ/Δ oocytes than in the oocytes from adult females, and that this causes juvenile Smc3Δ/Δ zygotes to develop more normally. However, to make this conclusion stronger, they should test whether expression of exogenous SMC3 protein can rescue the developmental arrest in Smc3Δ/Δ zygotes in adult females.
2) Along similar lines, the author should analyze the changes in SMC3 protein level from the juvenile to sexually mature stages. It would also be informative to determine when paternal Smc3 mRNA/protein is expressed after fertilization.
3) How did the authors get the zygote from juvenile females? Breeding would be difficult because of their small body size. If the zygotes were obtained by in vitro fertilization (IVF), the author should describe it in more detail in Results and also in Methods sections. Furthermore, if so, the success rate of IVF should also be stated.

Minor points:
Page 8, line 173: It is better to insert these two references at the end of the previous sentence.
Page 26, lines 558, 568, and 570: Please include a space before the unit of quantity.
Page 29, line 637: Does [#1.5] mean thickness?
Page 38, line 917: [Zp3-Crefemale] should be [Zp3-Cre female].
Throughout the manuscript:
The authors need to accurately describe the oocytes used in each experiment as GV oocyte, MII oocyte, etc.
Please unify the description with either live-cell (lines 249, 267, 274, 520 and 633) or live cell (lines 276, 356, 668, and 965).
Part of the references was garbled: Cahoon et al., 2017, Deehan et al., 2006 and Zheng et al., 2009.
Figures/legends:
Fig. 4B: Please use the same font size of the Y-axis values; zero appears to be smaller.
Fig. S6: [0.68] and [0.59] should be [p=0.68] and [p=0.59], respectively, as with the other figures.
Legend for Fig. S6: C57BL/6 represents mouse strain: not in italics. Also, isn't it unnecessary to describe [*, p < 0.05. ***]?

First revision

<u>Author response to reviewers' comments</u>

From the Editor:
I have now received all the referees' reports on the above manuscript, and have reached a decision. All three reviewers agree that the study provides novel insights into the role of SMC3 during embryonic development and potentially as a maternal effect gene. However, the referees have some significant concerns that will need to be addressed before we can consider publication. Specifically, as reviewer 1 suggests, the role of Smc3 upon deletion with Gdf9-Cre requires more substantiation. In line with this point, an analysis of the Smc3 with immunofluorescence to assess the localization to chromosomes would be more powerful than a bulk Western Blot which assesses total protein load. Reviewer 1 also suggests analysis of oocyte numbers directly with histology to definitively assess the impact of Smc3 deletion on ovarian reserve. Reviewer 3, similarly suggests a direct assessment of the claim that Smc3 functions as a maternal effect gene by experimentally rescuing the developmental arrest phenotype. Reviewer 2 provides suggestions to improve the

© 2021. Published by The Company of Biologists under the terms of the Creative Commons Attribution License (https://creativecommons.org/licenses/by/4.0/).

manuscript textually and to increase the scholarliness of the work. If you are able to revise along the lines suggested by the reviewers, I would be happy to receive a revision.
Our responses (in green):

We are thankful for all the reviewers' and editor's suggestions. We have revised the manuscript according to the constructive feedback. We have also modified the size of the figures to fit the journal's requirements. We highlight five main points which the editor had specifically requested we address, and then include a point by point response below.

1. We have added substantial new information regarding Gdf9-icre. We believe that since these results echo the Zp3-cre driver that they help provide experimental replication and confidence in the overall outcome.
2. The editor and reviewers requested that we examine SMC3 with immunofluorescence. This is an experiment that we attempted prior to submission. We are unable to complete this task because we cannot find a suitable antibody, despite trying several. The antibody we used in the manuscript only works for Western blotting, and not immunofluorescence. In fact we cannot find a convincing example of this experiment performed in the literature, so we imagine many people have found it challenging.
3. We added a new figure (Figure S3) that presents an analysis of oocyte numbers in histological sections to study the impact of loss of Smc3 on ovarian reserve with Zp3-cre and Gdf9-icre drivers.
4. We added a new figure and a table (Figure 5 and Table 3) demonstrating that microinjected Smc3 mRNA can rescue the 2-cell arrest phenotype, consistent with Smc3 actine as a maternal effect gene.
5. We have edited the manuscript throughout according to the reviewers' suggestions.

Reviewer 1 Advance summary and potential significance to field
This is an interesting manuscript from Yueh et al. that evaluates the requirement for the cohesin subunit Smc3 during meiosis and early embryogenesis. They take a mouse oocyte knockout approach to tackle this biological problem. Unlike deletion of other cohesin subunits, they find that meiosis in oocytes lacking Smc3 is normal, but there are significant abnormalities in the early embryo leading to a 2 cell embryo arrest and sterility. They very nicely detail accumulation of DNA damage after the 1st S phase in the embryo, accumulation of micronuclei, lagging chromosomes and loss of cohesion. Finally, they document that some of these defects depend on maternal age, as juvenile females do not have a 2 cell arrest, but do have some damage accumulation and appearance of abnormal chromosome configuration indicating loss of cohesion occurring.

Reviewer 1 Comments for the author
There are many aspects of this story that I find strong and are exciting findings (ie- the ones I just described). However, there are parts that are not strong and detract from the story that I detail below.

1. Use of Gdf9-Cre: The authors begin the MS by comparing KO of Smc3 using 2 Cre drivers and attempt to demonstrate that the Gdf9 Cre is excising Smc3 during meiotic S phase. The data presented is confusing and not convincing to this reviewer. This is because the authors show 2 BGal stained gonads with varying expression levels (1 high, 1 low), suggesting that the expression of Gdf9 at this time must vary and is likely mosaic. How many gonads were weak vs strong expression in their sampling? Do all the oocytes in the gonads express the Cre? They should document that SMC3 is being removed at this time. They also mention 2 time points of S phase; e13.5 and e11.5. Which is it? I thought it was the earlier time point? If the timing of S phase is not clear, and if it is not clear that all oocytes are excising Smc3 at this time, then it is not convincing that the Gdf9 model is really that different in biological timing than the Zp3. Then, this model is not used for the rest of the MS because the phenotype is the same as the Zp3 model. So, I find use of this model and the time spent building this part of the MS distracting. I strongly recommend removing it; the paper will be improved without it. If the authors elect to keep it, they will need to do several more experiments to make it convincing for something that overall does not contribute to the main point of the MS.
We thank the reviewer for these suggestions and the opportunity to clarify. We now provide the number of β-Gal gonads in each genotype with varying expression levels in Fig S1. In brief, 4 of 6 R26R Gdf9-iCre+ female gonads were weakly positive and 2 of 6 display strong expression.

© 2021. Published by The Company of Biologists under the terms of the Creative Commons Attribution License (https://creativecommons.org/licenses/by/4.0/).

Oocytes enter S phase at e13.5. Because Gdf9-iCre is expressed at e13.5 (Fig. S1), we can confirm that the cre is induced at e13.5 and therefore we expect a depletion in protein, which was confirmed by Western blotting in GV oocytes at e13.5 (Figure S2, source data). We have now corrected the typo e11.5 to e13.5 in figure S1B.
We think that by providing new results regarding the Gdf9-icre driver that we have built replication into the experimental design rather than distraction.

2.Calling this strain a KO: The level of KO via western blotting is concerning. The quantification shows that there is still about 20% of the protein remaining in GV oocytes, which makes it a depletion, not a knockout. If they were to collect MII eggs, would the deletion be better (due to turnover during MI) and therefore explain why the embryo is impacted and not the oocyte? The reason why it concerns me is because the authors make the claim that it is not required for meiosis. But, formally, this cannot be stated because it is not a true KO. It is possible that the remaining 20% supports MI but not the embryo or it is possible that by Met II there is much less than 20% explaining why the embryos are impacted but not the oocyte. Therefore, the claim that this is a maternal effect gene is over-interpreted. Another possibility is that there is no Smc3 loaded, and the 20% is cytoplasmic and not functional. Can the authors try performing IF for SMC3 during MI? The functional population is much more important and would be more convincing as a maternal effect gene if the remaining is simply not at the right place (ie- not functional). Alternatively, the authors could try siRNA or Trim-Away depletion and support the claim of not being required during MI.
We thank the reviewer giving us this opportunity to clarify the Smc3 cKO strain. The reviewer makes an excellent point about gene deletion vs protein depletion and an excellent suggesting regarding IF to assess the functional pool of Smc3 protein. Prior to submission, we tried to detect Smc3 by IF but the antibodies were not satisfactory. The Smc3 gene is conditionally knocked out by two different drivers, with similar outcomes. The conditional knockout leads to depletion of the protein. In fact we believe that Smc3 is likely required for meiosis but the low levels present after the conditional knockout suffice for M1 and M2. This is now stated more explicitly.
3.Age: Can the authors explain why 2 weeks makes a difference in the 2 cell arrest phenotype? Does this reflect a difference on hormonal impact of oocytes that are not ovulated in the 1st wave?
We thank the reviewer bringing up this interesting question. In fact, we don’t know the reason why a short 2-week time window can make a dramatic difference in the 2-cell arrest phenotype. It is possible that hormones impact the oocytes that are not ovulated in the 1st wave. More experiments will be needed to address this question, but they lie outside the scope of this manuscript.

4.Reserve: The table 2 indicates fewer zygotes isolated from KOs. Do they ovulate fewer eggs or have fewer GV oocytes? If so, maybe the oocytes impacted by reduction of Smc3 are removed from the ovarian reserve during growth? These basic parameters should be evaluated because the authors could be missing an important part of the phenotype. This is because the authors claim only an effect on the embryos, but perhaps they are missing the effect on oocytes because they die.
The reviewer makes an excellent point. We now provide data regarding ovarian reserve in Figure S3. Although there are more mature follicles in the mutant for reasons we do not understand, the overall ovarian reserve is very similar, so a low ovarian reserve does not explain the differences observed.

5.Western: The authors should show the full length images of westerns.
We thank the reviewer’s suggestion. Now in the supplement information, we add a new page called “Source Data” to show all the full-length images of Western blots.

6.A few comments in the text I recommend changing:
Line 64: “How genome integrity at this stage is monitored by checkpoints is an open question” I would modify because the authors don’t address any checkpoints.
All references to checkpoints have been deleted in the intro, although we think our data suggests that there is not a strong checkpoint operating at the first mitosis in the zygote that would catch chromosome misattachment and segregation.

Line 90- also brings in checkpoints, but really this isn’t a checkpoint paper so I find it confusing
All references to checkpoints have been deleted in the intro.
Line 122- also brings up a checkpoint- same confusion.
All references to checkpoints have been deleted in the intro.

© 2021. Published by The Company of Biologists under the terms of the Creative Commons Attribution License (https://creativecommons.org/licenses/by/4.0/).

Line 95- mentions “the same Zp3-cre driver” but this is the first time that is mentioned. Needs to be edited.
We thank the reviewer for his/her suggestion. We have removed “same” in this sentence.

Line 130- How is the Cre improved? This is the standard cre to my knowledge.
We thankful the reviewer for pointing out this issue. The Gdf9-iCre is an improved Cre recombinase and Zp3-Cre is a standard Cre. We apologize for our mistake and now have revised the entire manuscript text and figures accordingly.

Line 151- describes the het levels being intermediate. The graph shows Smc3 levels being closer to KO so I find this point not accurate.
We agree with the reviewer’s comment. Now we have rewritten the sentence as “The level of SMC3 in heterozygotes is comparable to cKO and significantly lower than WT.”

Figure 1B- are these MI or MII spindles?
Yes, these are MII spindles, now specified.
Line 301- I think you mean parental, not paternal.
We are grateful that the reviewer raised this question. In fact, we mean the paternal genome. Because Smc3 has been deleted in the oocyte, we were making the point that once the paternal Smc3 gene is expressed in the late 2-cell stage, the resulting protein could enable embryogenesis.

Reviewer 2 Advance summary and potential significance to field
The study provides novel insight into the working mechanism of maternal Smc3 in early embryonic development. Using a plethora of cutting-edge experimental techniques, the research demonstrates that oocyte-stored SMC3 is required to support the integrity of the zygotic genome during the very first round of DNA replication and sister chromatid segregation to successfully pass through the first and second mitotic divisions in the embryo, making cohesin a key protector of the zygotic genome.

Reviewer 2 Comments for the author
1. Please provide full forms of all abbreviations when first used in manuscript body and abstract. This would also help non-expert readers.
We thank the reviewer for this suggestion. We have now added the full forms of all abbreviations when first used in manuscript body and abstract as following:
Abstract: line 27- Structural maintenance of chromosomes protein 3 (SMC3)
Manuscript: p4 line 90- CCCTC-binding factor (CTCF), p7 line 139- germinal vesicle (GV) stage, and p12 line 280- anaphase-promoting complex/cyclosome (APC/C).

2. Please specify the number of replicates conducted for each method, and if results were consistent across all replicates. This is important to ensure reproducibility.
We thank the reviewer for the kind reminder to specify the number of replicates conducted for each method, so that we can clarify for the reader the robustness of the data. In the revised manuscript, we add the number of oocytes, embryos, and mice we used for each experiment in the main text, figures, and figure legends.

3. Please double-check any minor typos/errors in spelling, punctuation, grammar, etc. (for e.g., Western and not 'western' blot, use 'z' or 'Z' confocal image-stacks consistently, etc.).
We are grateful for this reminder. We have now corrected the typo of “Western blot”, “Z-stack”, “Z-projection” throughout.

4. In the abstract and at the end of conclusions, it would be great to include the overall, big-picture application and significance of the work.
We are grateful for the reviewer’s suggestion. We now add one brief sentence in the abstract and at the end of the discussion to highlight significance. The first point is that despite previous reports of aneuploidy in early embryos, chromosome missegregation in zygotes halts embryogenesis at the 2-cell stage. The second point is that elongated spindles in zygotes and micronuclei at the 2-cell stage could serve as visual markers indicated poor embryonic outcomes, which could be useful for IVF.

© 2021. Published by The Company of Biologists under the terms of the Creative Commons Attribution License (https://creativecommons.org/licenses/by/4.0/).

5. Please discuss any limitations of the study and recommend potential future directions to overcome them.
We thank the reviewer for the suggestion. Additional depletion strategies may help, along with methods to determine the functional pool, and a broader examination of spindle function, all now stated in the discussion.

6. Methods - Statistics - please include Fishers exact test suitably.
We are grateful to reviewer's suggestion. we have included Fisher's exact test in the Methods-Statistical session. "Fisher's exact test was performed to examine the contingency table datasets."

7. Figures 3B and 6C - is it possible to specify the units for the intensities?
We are grateful to this suggestion and now add arbitrary units (a.u.) for the intensity in Fig 3B. The y axis is the normalized intensity that the value is divided to the highest intensity value, so it doesn't have unit.

8. Please double-check and clarify all relevant controls for each of the methods.
We appreciate the reviewer's reminder. We have double-checked and clarified the relevant controls for each experiment throughout the manuscript.

9. To increase the cross-domain visibility and citability of this paper, additional works on some relevant conserved proteins like the 14-3-3 (YWHA), which are known to be abundant in mammalian oocytes and and eggs (including meiotic spindle) and interact isoform-specifically with multiple other factors to regulate molecular cross-talks in oocyte maternal effects, developmental competence, oocyte maturation, and/or early embryogenesis, could be suitably implicated by citing the following:
a) https://link.springer.com/article/10.1186/1756-0500-5-57
b) https://doi.org/10.1186/1471-213X-13-10
c) https://link.springer.com/article/10.1186/s12861-019-0200-1
d) https://www.tandfonline.com/doi/pdf/10.4161/cc.24991

Thankyou for the reading material! We appreciate the recommendation to broaden the scope of the discussion and have incorporated two of these references into the discussion.

Reviewer 3 Advance summary and potential significance to field
In this manuscript, Yueh et al. found that maternal SMC3 protein is required for maintain of the zygotic integrity and its chromatin segregation, and that depletion of maternal Smc3 gene results in developmental arrest at the 2-cell stage (although such phenotype is dependent on maternal age). Overall, the characterization of SMC3 as a maternal effect gene is significant advance to the field of mammalian embryonic development. Moreover, most data is of good quality, and the experiments are clearly and logically presented. However, there are several aspects of the manuscript that need to be improved/strengthened as follows:

Reviewer 3 Comments for the author
Major points:

1) My main concern is about the developmental ability of zygotes collected from juvenile (3-4 weeks old) Smc3Δ/Δ female. The authors argue that there is more SMC3 protein remaining in the juvenile Smc3Δ/Δ oocytes than in the oocytes from adult females, and that this causes juvenile Smc3Δ/Δ zygotes to develop more normally. However, to make this conclusion stronger, they should test whether expression of exogenous SMC3 protein can rescue the developmental arrest in Smc3Δ/Δ zygotes in adult females.
We are thankful for the reviewer's suggestion. We performed a microinjection rescue experiment and added a new figure (Figure 5) with the results. In brief, we microinjected the in vitro transcribed Smc3 mRNA into the Smc3 mutant zygotes. Our results suggest that exogenous SMC3 can rescue the developmental arrest in Smc3Δ/Δ zygotes in adult females and post-injection we observe embryos that can now mature past the 2-cell stage.

2) Along similar lines, the author should analyze the changes in SMC3 protein level from the juvenile to sexually mature stages. It would also be informative to determine when paternal Smc3 mRNA/protein is expressed after fertilization.

© 2021. Published by The Company of Biologists under the terms of the Creative Commons Attribution License (https://creativecommons.org/licenses/by/4.0/).

We provided Western blot analysis of Smc3 protein levels in GV oocytes derived from juvenile versus sexually mature females. Because zygotic genome activation occurs at the late 2-cell stage in mouse embryos (Schultz 1993, 2002), we expect paternal Smc3 mRNA/protein will be expressed at the 2-cell stage. However, paternal mRNA cannot be distinguished from maternally loaded mRNA.

3) How did the authors get the zygote from juvenile females? Breeding would be difficult because of their small body size. If the zygotes were obtained by in vitro fertilization (IVF), the author should describe it in more detail in Results and also in Methods sections. Furthermore, if so, the success rate of IVF should also be stated.
We appreciate the reviewer giving us this opportunity to explain the methodology of acquiring zygotes from juvenile females. As we mentioned in the manuscript, a breeding trial is not feasible for the juvenile females. Therefore, we acquired zygotes from the juvenile females by superovulation. In brief, we inject PMSG following HCG to stimulate juvenile females to ovulate oocytes and collect zygotes following mating. IVF was not used.

Minor points:

Page 8, line 173: It is better to insert these two references at the end of the previous sentence.
We appreciate the reviewer's suggestion. We have now inserted (Hamatani et al., 2004, Aoki et al., 1997) at the end of the previous sentence.
Page 26, lines 558, 568, and 570: Please include a space before the unit of quantity.
We are very grateful to the reviewer for pointing out the typos. We have now added a space before the unit of quantity.
Page 29, line 637: Does [#1.5] mean thickness?
#1.5 is the number of the coverslip which is 0.17 mm in thickness.
Page 38, line 917: [Zp3-Crefemale] should be [Zp3-Cre female].
We are thankful for the reviewer's comment. We have now fixed that typo.

Throughout the manuscript:
The authors need to accurately describe the oocytes used in each experiment as GV oocyte, MII oocyte, etc.
Thankyou for allowing us to clarify this point for each experiment. We double-checked the description of the oocyte used in each experiment throughout the manuscript. Most of the missing information was for GV stage oocytes. In figure S2 and S6, we have now clearly stated that GV stage oocytes were used in both experiments. We have indicated stage in the manuscript text throughout.
Please unify the description with either live-cell (lines 249, 267, 274, 520, and 633) or live cell (lines 276, 356, 668, and 965).
We thank the reviewer for pointing out the inconsistency. We have now unified all the description, using live-cell throughout the manuscript.
Part of the references was garbled: Cahoon et al., 2017, Deehan et al., 2006, and Zheng et al., 2009.
We have now fixed the errors in the references.
Figures/legends:
Fig. 4B: Please use the same font size of the Y-axis values; zero appears to be smaller.
We appreciate the reviewer's comment. The font size of the Y-axis values in Fig. 4B are all the same size now.
Fig. S6: [0.68] and [0.59] should be [p=0.68] and [p=0.59], respectively, as with the other figures.
We are grateful that the reviewer pointed out this issue. We have now changed the description to [p=0.68] and [p=0.59] as with other figures.
Legend for Fig. S6: C57BL/6 represents mouse strain: not in italics. Also, isn't it unnecessary to describe [*, p < 0.05. ***]?
We are grateful for the reviewer's suggestions. We have changed "C57BL/6" to regular font. We agree that most biologists are familiar with * as p value < 0.05 and *** as < 0.001. However, most journals prefer to include a numeric value.

© 2021. Published by The Company of Biologists under the terms of the Creative Commons Attribution License (https://creativecommons.org/licenses/by/4.0/).

Second decision letter

MS ID#: DEVELOP/2021/199800

MS TITLE: Maternal Smc3 protects the integrity of the zygotic genome through DNA replication and mitosis

AUTHORS: Wei-Ting Yueh, Vijay Pratap Singh, and Jennifer Gerton

I have now received all the referees reports on the above manuscript, and have reached a decision. The overall evaluation is positive; the reviewers recommend clarifications to the text of the manuscript to enhance the rigor in the analysis and clarity. Specifically Reviewer 1 asks for greater discussion of the difference in phenotypes between the Gdf9 vs the Zp3 mouse with context to the assays used in the study and for ackoweldegement and a potential discussion on the low level of rescue (22%) upon injection of Smc3. Having looked at the study myself, I agree with the need for further discussion of both these points. Reviewer 3 asks for greater clarification in the methods used to perform super ovulation in young females, more with respect to the feasibility of this mating in terms of litter sizes etc. Please attend to all of the reviewers' comments in your revised manuscript and detail them in your point-by-point response. If you do not agree with any of their criticisms or suggestions explain clearly why this is so.

Reviewer 1

Advance summary and potential significance to field

Similar to my first assessment, there are a lot of important findings reported in this manuscript. I have some minor comments below that could be handled by text edits.

Comments for the author

1. Gdf9-iCre: Although I appreciate the extra work included on this line in the revision, I still find its inclusion problematic. I respect that it helps validate that depletion of Smc3 causes sterility. However, the authors now make a significant point to demonstrate that Smc3 is deleted during pre-meiotic S phase, a time when cohesin is being deposited. This is biologically distinct from when Zp3-Cre would act. Therefore, this point makes me want to know more specifics of what is different phenotypically about the Gdf9 mouse vs the Zp3 mouse in the assays that they show. I find the MS substantial on its own without inclusion of this information and still recommend its removal, especially now that the new information makes the lack of phenotypic follow up unsatisfying.
2. Smc3 rescue: The authors added new data rescuing the cell cycle arrest of 2 cell embryos by injecting Smc3 into depleted zygotes. They report a 22% rescue (9/44 zygotes). This is a quite a low percentage for a rescue and this point is not addressed. Why do the authors think the rescue is so poor? Are they confident that the protein is being translated in all 44 zygotes? Are they confident that all zygotes are still pre-S phase when injected? The authors must comment on this in the text. Alternatively, the authors could consider injected oocytes or eggs and activating the eggs to see if depositing the protein in the egg makes are more significant rescue.
3. Page 6-7: I recommend that the authors include the level of depletion from the western blots in the text for ease of readership since the data is supplemental and won't be in the body of the publication.
4. Line 139: “completely sterile” is redundant. The animals are sterile (there is no incomplete sterile!). Please edit.
5. Page 7: In the new histology, the authors should comment on the presence of corpus lutea to further support that these animals ovulate without administration of hCG.
6. Figure 1D: How many animals were examined? An n of 11 oocytes is a very small number if multiple animals were used. A standard in the field is to examine oocytes from at least 3 animals, and ideally 5.
7. Figure 1C: I recommend changing the blue color for the chromatids to gray so that more features can be visualized in the print.

© 2021. Published by The Company of Biologists under the terms of the Creative Commons Attribution License (https://creativecommons.org/licenses/by/4.0/).

8. Line 326: The authors state that they test if levels of Smc3 in juveniles can “rescue” the DNA lesions. I find the use of “rescue” here odd since you are really just assessing the phenotype in young animals that happen to have physiologically WT protein levels. Can the authors reword this sentence for clarity?
9. Lines 500-502: Some important aging/cohesion papers that led to the Duncan 2012 study are not included in the citations:
https://pubmed.ncbi.nlm.nih.gov/20817534/, https://pubmed.ncbi.nlm.nih.gov/20817533/
https://pubmed.ncbi.nlm.nih.gov/20971813/

Reviewer 2

Advance summary and potential significance to field

The study provides novel insight into the working mechanism of maternal Smc3 in early embryonic development. The results suggest elongated spindles in zygotes and micronuclei in the 2-cell embryo are visual markers of poor developmental outcomes which could be useful for IVF.

Comments for the author

Thank you for the revisions.

Reviewer 3

Advance summary and potential significance to field

The authors made precise revisions according to my comments. I was satisfied with the results, especially in the rescue experiments, which are very difficult, and the results are very good. However, I have one regret about the following points which I would like to be addressed.

Comments for the author

I routinely collect oocytes and fertilized eggs from mice, but I have no experience in collecting fertilized eggs by mating such juvenile (3-4 wk) female mice with male mice. The juvenile females used in this study (B6 background) are thought to have a small body size (even if hormones are used to induce ovulation), so I wondered if it would be possible to mate them. In addition, when the hormones are administered to juvenile female mice, a significant number of oocytes (nearly dozens in the case of B6 mice) are generally ovulated. Under these conditions, it is unlikely that all oocytes will be fertilized, even if the male mice are successfully mated and fertilized. I make this comment because the results of experiments with oocytes derived from juvenile female mice play an important part. It would be even better to have detailed information on what percentage of the eggs collected the day after mating were fertilized.

Second revision

Author response to reviewers' comments

The overall evaluation is positive; the reviewers recommend clarifications to the text of the manuscript to enhance the rigor in the analysis and clarity. Specifically Reviewer 1 asks for greater discussion of the difference in phenotypes between the Gdf9 vs the Zp3 mouse with context to the assays used in the study and for acknowledgement and a potential discussion on the low level of rescue (22%) upon injection of Smc3. Having looked at the study myself, I agree with the need for further discussion of both these points. Reviewer 3 asks for greater clarification in the methods used to perform super ovulation in young females, more with respect to the feasibility of this mating in terms of litter sizes etc. Please attend to all of the reviewers' comments in your revised

© 2021. Published by The Company of Biologists under the terms of the Creative Commons Attribution License (https://creativecommons.org/licenses/by/4.0/).

manuscript and detail them in your point-by-point response. If you do not agree with any of their criticisms or suggestions explain clearly why this is so.

Dear Editor-

Thankyou for your continued attention to our manuscript. We have made edits based on the comments of the reviewers and yourself and hope you will now find the manuscript ready for publication.

Summary of changes:

1) The color in Figure 1 was edited for better visualization
2) Corpus lutea are highlighted in Figure S3
3) Edits were made to the text and figure legends to add clarity, citations, information about drivers, experimental replication, and microinjection

Reviewer 1 Advance Summary and Potential Significance to Field:
Similar to my first assessment, there are a lot of important findings reported in this manuscript. I have some minor comments below that could be handled by text edits.

Reviewer 1 Comments for the Author:
1.Gdf9-iCre: Although I appreciate the extra work included on this line in the revision, I still find its inclusion problematic. I respect that it helps validate that depletion of Smc3 causes sterility. However, the authors now make a significant point to demonstrate that Smc3 is deleted during pre-meiotic S phase, a time when cohesin is being deposited. This is biologically distinct from when Zp3-Cre would act. Therefore, this point makes me want to know more specifics of what is different phenotypically about the Gdf9 mouse vs the Zp3 mouse in the assays that they show. I find the MS substantial on its own without inclusion of this information and still recommend its removal, especially now that the new information makes the lack of phenotypic follow up unsatisfying.

We appreciate the reviewer giving us the chance to discuss the rationale of including Gdf9-icre data. We made the point that Gdf9-icre acts during pre-meiotic S phase in our initial submission, so we would like to first state that this timing is not a new point. The timing difference between the two cre drivers is what initially drove us to use them both. However, because Smc3 protein persists after gene deletion, the phenotype takes time to manifest. We favor the explanation that the Gdf9-cre oocytes have sufficient Smc3 to complete meiosis, not the alternative explanation that Smc3 is not needed for meiosis. The two cre deletion programs **do not show any difference** in fertility, meiosis, or embryogenesis. The phenotypic follow up was extensive and we respectfully disagree that it is unsatisfying and should be removed. Our opinion is that the findings support the overall conclusion that depleted levels of Smc3 in the oocyte are sufficient to support meiosis, but maternally loaded Smc3 is critical for embryogenesis. We have modified the text for clarity (p 7, line 137, p 9, line 171).

2.Smc3 rescue: The authors added new data rescuing the cell cycle arrest of 2 cell embryos by injecting Smc3 into depleted zygotes. They report a 22% rescue (9/44 zygotes). This is a quite a low percentage for a rescue and this point is not addressed. Why do the authors think the rescue is so poor? Are they confident that the protein is being translated in all 44 zygotes? Are they confident that all zygotes are still pre-S phase when injected? The authors must comment on this in the text. Alternatively, the authors could consider injected oocytes or eggs and activating the eggs to see if depositing the protein in the egg makes are more significant rescue.

We appreciate the reviewer giving us the opportunity to discuss the rescue experiment. There are several factors that make this experiment quite challenging, technically and experimentally, but we note that reviewer 3 commented that the rescue was “very good”. We performed the microinjection at ~22 hour-post-hCG injection (hpi) because we determined that 24 dpi is when S phase typically occurs based on the Click-it assay (see Figure 2). We cannot confirm the SMC3 protein is being translated in all zygotes. However, we validated the microinjection protocol by injecting mCherry mRNA into zygotes and all the injected embryos were mCherry positive at the 2-cell stage (not shown). We have now added comments in the text (p14 line 313) to explain the potential sources of the imperfect rescue, but we think that the rescue observed in a subset of

© 2021. Published by The Company of Biologists under the terms of the Creative Commons Attribution License (https://creativecommons.org/licenses/by/4.0/).

zygotes supports the idea that sufficient SMC3 provided in the correct time window can enable developmental competence.

3.Page 6-7: I recommend that the authors include the level of depletion from the western blots in the text for ease of readership since the data is supplemental and won't be in the body of the publication.
We are grateful to the reviewer's kind suggestion. We now add the level of SMC3 depletion from the western blots at page 6 line 134 and page 7 line 144.

4.Line 139: "completely sterile" is redundant. The animals are sterile (there is no incomplete sterile!). Please edit.
We thank the reviewer's kind reminder and have deleted "completely".

5.Page 7: In the new histology, the authors should comment on the presence of corpus lutea to further support that these animals ovulate without administration of hCG.
We thank the reviewer for his/her suggestion. We added a comment on the presence of corpus lutea at page 7 line 151, which help to rule out lack of ovulation as a mechanism of infertility. In addition, we marked corpus lutea in Figure S3.

6.Figure 1D: How many animals were examined? An n of 11 oocytes is a very small number if multiple animals were used. A standard in the field is to examine oocytes from at least 3 animals, and ideally 5.
We appreciate the opportunity to clarify our sample sizes in Figure 1. Although many oocytes were collected from several animals, n=44 from 6 animals for the *Smc3*$^{fl/fl}$ and n=24 from 5 animals for the *Smc3*$^{fl/fl}$*;Gdf9-iCre* (p 39, line 982) we only claim to have assessed a subset of these cases in Figure 1B, because the spindle needs to be in a favorable orientation to determine normality. This is the most conservative scoring method. We have also added the animal number for Figure 1C-D in the figure legend at page 39 line 988.

7.Figure 1C: I recommend changing the blue color for the chromatids to gray so that more features can be visualized in the print.
We thank the reviewer for this suggestion. We changed the color of chromatids to gray but the centromere could not be well-visualized. To solve the issue, we instead changed the chromatid channel to magenta and the CREST channel to green for better visualization. In addition, we added zoomed images for individual chromatid pairs to highlight the chromosome structure. We hope those changes help the reader visualize our results.

8.Line 326: The authors state that they test if levels of Smc3 in juveniles can "rescue" the DNA lesions. I find the use of "rescue" here odd since you are really just assessing the phenotype in young animals that happen to have physiologically WT protein levels. Can the authors reword this sentence for clarity?
We appreciate the reviewer pointing out a bad word choice. We have now reworded the sentence ("rescue" to "were sufficient") at page 15 line 337.

9.Lines 500-502: Some important aging/cohesion papers that led to the Duncan 2012 study are not included in the citations:
https://pubmed.ncbi.nlm.nih.gov/20817534/,https://pubmed.ncbi.nlm.nih.gov/20817533/
https://pubmed.ncbi.nlm.nih.gov/20971813/

Thankyou for pointing out these omissions in our reference list. We now include them in our citations in the discussion, page 23 line 511.

Reviewer 2 Advance Summary and Potential Significance to Field:
The study provides novel insight into the working mechanism of maternal Smc3 in early embryonic development. The results suggest elongated spindles in zygotes and micronuclei in the 2-cell embryo are visual markers of poor developmental outcomes, which could be useful for IVF.

© 2021. Published by The Company of Biologists under the terms of the Creative Commons Attribution License (https://creativecommons.org/licenses/by/4.0/).

Reviewer 2 Comments for the Author:
Thank you for the revisions.

Reviewer 3 Advance Summary and Potential Significance to Field:
The authors made precise revisions according to my comments. I was satisfied with the results, especially in the rescue experiments, which are very difficult, and the results are very good. However, I have one regret about the following points, which I would like to be addressed.

Reviewer 3 Comments for the Author:
I routinely collect oocytes and fertilized eggs from mice, but I have no experience in collecting fertilized eggs by mating such juvenile (3-4 wk) female mice with male mice. The juvenile females used in this study (B6 background) are thought to have a small body size (even if hormones are used to induce ovulation), so I wondered if it would be possible to mate them. In addition, when the hormones are administered to juvenile female mice, a significant number of oocytes (nearly dozens in the case of B6 mice) are generally ovulated. Under these conditions, it is unlikely that all oocytes will be fertilized, even if the male mice are successfully mated and fertilized. I make this comment because the results of experiments with oocytes derived from juvenile female mice play an important part. It would be even better to have detailed information on what percentage of the eggs collected the day after mating were fertilized.

We thank the reviewer for giving us the opportunity to discuss the protocol for collecting fertilized eggs from juvenile female mice. The juvenile females are in fact smaller than adult females. However, the 3-6-week-old juvenile females ovulate in response to external hormones. The female mice mate with males following PMSG/hCG administration. We essentially followed the protocol from the *Manipulating the Mouse Embryo* textbook and JAX laboratory (Superovulation technique (jax.org)), which recommends this procedure to superovulate juvenile females to collect time mated embryos. This is one reason why we find the juvenile mutant results interesting and emphasize this finding in the discussion (page 23 line 520). We added a short comment that analysis of embryos from juveniles is recommended in some textbooks. Given that this is a standard protocol for assessing embryogenesis, others may use it, and could have erroneously concluded that their gene of interest is not a maternal effect gene if they only analyzed zygotes from juveniles. We did not keep detailed information on the percentage of eggs that were fertilized.

Third decision letter

MS ID#: DEVELOP/2021/199800

MS TITLE: Maternal Smc3 protects the integrity of the zygotic genome through DNA replication and mitosis

AUTHORS: Wei-Ting Yueh, Vijay Pratap Singh, and Jennifer Gerton
ARTICLE TYPE: Research Article

I am happy to tell you that your manuscript has been accepted for publication in Development, pending our standard ethics checks.

© 2021. Published by The Company of Biologists under the terms of the Creative Commons Attribution License (https://creativecommons.org/licenses/by/4.0/).
